# Mapping terrestrial macroplastics and polymer-coated materials in an urban watershed using WorldView-3 and laboratory reflectance spectroscopy

**DOI:** 10.1007/s10661-025-14125-z

**Published:** 2025-06-25

**Authors:** Elena Aguilar, Daniel Sousa, Amy V. Uhrin, Napoleon Gudino-Elizondo, Trent Biggs

**Affiliations:** 1 Department of Geography, San Diego State University, San Diego, CA 92182 USA; 2 National Oceanic and Atmospheric Administration, National Ocean Service, Office of Response and Restoration, Marine Debris Division, Silver Spring, MD 20910 USA; 3 Instituto de Investigaciones Oceanológicas, Universidad Autónoma de Baja California, 22760 Ensenada, Baja CA Mexico

**Keywords:** Terrestrial anthropogenic debris, Urban watershed, Macroplastics, Reflectance spectroscopy, WorldView-3, Matched filter

## Abstract

The presence and abundance of synthetic polymers can be used to map mismanaged waste, housing quality, and other development indicators. For various common urban plastic polymers, spectral absorption features in the shortwave infrared (SWIR) wavelengths can be captured using laboratory reflectance spectroscopy. In addition, SWIR bands of the WorldView-3 (WV3) satellite sensor are known to capture absorptions specific to these polymers. The ≈4-m ground sampling distance of WV3 offers the potential to spatially resolve urban aggregations of polymer-based debris and differentiate these from natural and other anthropogenic materials. Synthesizing field work, laboratory reflectance spectroscopy, and satellite image analysis, we mapped suspected polymer-based materials in a complex urban watershed on the coastal US-Mexico border. Field surveys identified several large (80–150 m^2^) unmanaged waste aggregations in stream channels. 1024-band laboratory reflectance spectra of field-collected macroplastics (25-1000 mm) showed consistent absorption features that were often preserved when convolved to the 8-band spectral response function of the WV3 SWIR sensor. Laboratory spectra for high- and low-density polyethylene, polyethylene terephthalate, and polyvinyl chloride correlated closely (*r* = 0.95; *p* < 0.01) and had high spectral similarity with an independently derived endmember selected from a bounding apex of the spectral feature space of a WV3 image of the area. Matched filtering identified areas corresponding to infrastructure inferred to be of polymer-based or -coated materials as well as waste aggregations. Mapping precision scores ranged from 0.92 to 0.95. Our study suggests that some common plastic polymers may be distinguishable in this study area using a generalized WV3 SWIR image endmember.

**Supplementary Information:**

The online version contains supplementary material available at 10.1007/s10661-025-14125-z.

## Introduction

Plastic waste from land-based sources contributes substantially to oceanic plastic pollution whether through riverine transport and outflow (Lebreton et al., 2017, Schmidt et al., 2017, Meijer et al., 2021) or via direct deposition of mismanaged plastic waste at the coast (Jambeck et al., 2015). Important knowledge gaps remain with regard to plastic distribution and loading from terrestrial sources, particularly from watersheds in understudied countries (Blettler et al., 2018), which are often characterized by rapid urbanization, growing populations, flood hazards, and limited infrastructure investment and waste management resources. Informal landfills in such watersheds have an especially high risk of seeping and leaching waste constituents into soils, surface waterbodies, and groundwater resources (Goushki et al., 2023; Pandit & Kateja, 2023). The ability to accurately map aggregations of anthropogenic waste in watersheds is thus important for long-term monitoring efforts.

Monitoring efforts for terrestrial anthropogenic waste at watershed scales largely rely on time- and labor-intensive field surveys (Aslam et al., 2022; Cordova et al., 2023). Remote sensing has the potential to provide additional information, including characterization of the spatial patterns of informal landfills, information on potential sources, and estimation of the overall amounts of anthropogenic waste in a given study area (Martínez-Vicente et al., 2019; Guha et al., 2023). Urban waste presents particularly challenging problems in the spatial (submeter-to-meter scale features), spectral (potential for diverse polymer types), and temporal (potential for subdaily deposition and redistribution) realms. The accuracy of remotely sensed maps of anthropogenic waste tend to lack consensus due to a deficit of in situ data (Maximenko et al., 2019; Blume et al., 2023). Even so, maps of detected waste have the potential to assist local management by targeting monitoring and cleanup efforts on specific hotspots and expanding spatial and temporal monitoring (Kruse et al., 2023).

The terms “plastic” and “polymer” generally refer to a broad, extremely diverse set of chemicals, compounds, and materials (Geyer et al., 2017; Wagner et al., 2024). In global urban environments, the most common polymer types (excluding recycled and bio-based plastics) are petroleum-based and include polypropylene (PP), polyethylene terephthalate (PET), high- and low-density polyethene (HDPE, LDPE), polystyrene (PS), polyamide (PA), polyvinyl chloride (PVC), and polyurethane (PUR) (Geyer et al., 2017; PlasticsEurope, 2022). These polymers are also most abundant in freshwater systems (van Emerik & Schwarz, 2020). Differences and similarities in polymer structure among these plastic types can have implications for remotely sensed detection of urban plastics particularly the use of visible to shortwave infrared (VSWIR) reflectance (Garaba & Dierssen, 2018).

In the last 5 years, a number of studies have used laboratory reflectance spectroscopy to examine the spectral characteristics of plastic waste. These have included samples from dried or submerged (wet) samples of marine-harvested macroplastics, as well as samples retrieved from rivers and virgin plastics (Table 1). Many absorption features in different plastic polymer types remain consistent in the shortwave infrared (SWIR) spectrum despite color differences (Garaba et al., 2021). The generative physical processes for the specific absorption features are not fully understood, and are generally attributed to differences in covalent bond structure (Moshtaghi et al., 2021). SWIR absorption features exist near 931, 1130–1192, 1205–1215, 1394–1460, 1660, and 1728–1732 nm across several different types of macroplastic polymers harvested from various marine compartments (Garaba & Dierssen, 2018; Garaba & Dierssen, 2020; Moshtaghi et al., 2021; Tasseron et al., 2021; Knaeps et al., 2021). Schmidt et al. (2023) simulated various multispectral sensors including SWIR imagery of the WorldView-3 (WV3) commercial satellite, based on laboratory spectra of common plastic types, and identified that narrow SWIR channels with central wavelengths at 1141, 1217, 1697, and 1716 nm have the highest capability for plastic differentiation.

**Table 1:** Absorption features for polymers commonly found in macroplastics. Wavelengths labeled as “Consolidated” are shared absorptions that span the studied polymer types

Main polymer types	Absorption centers/ranges (nm)
Polypropylene (PP)	1192, 1217^6^, 1394, 1730^1^; 1205, 1400^2^, 1716^6^
Polyethylene terephthalate (PET)	1130, 1660^1^; 1415, 1660, 1908, 2132^5^
Low-density polyethylene (LDPE)	1192, 1394, 1730^1^; 1210, 1430^2^; 1206–1213, 1400–1430, 1728, 2308–2313^5^
High-density polyethene (HDPE)	1210, 1430^2^; 1206–1213, 1400–1430, 1728, 2308–2313^5^
Polystyrene (PS)	1150, 1450^2^; 1141^6^, 1678, 2167^5^
Polyester (PEST)	1130, 1413, 1660^1^
PET, LDPE, PEST, PP	Consolidated^3^: 1216, 1397, 1730
Polyvinyl chloride (PVC), Polyamide/nylon (PA), LDPE, PET, PP, PS	Consolidated^4^: 931, 1045, 1215, 1417, 1537, 1716^6^, 1732, 2046, 2313
PET, LDPE, HDPE, PS, PVC	Consolidated^5^: 1210, 1415, 1660, 1728, 2132–2313

^1^Moshtaghi et al., 2021; Method: Analytical Spectral Devices (ASD) FieldSpec4 spectroradiometer

^2^Tasseron et al., 2021; Method: Specim FX10 hyperspectral camera

^3^Knaeps et al., 2021; Method: ASD FieldSpec4 & Spectral Evolution spectroradiometers

^4^Garaba & Dierssen, 2018, 2020; Method: ASD FieldSpec4 spectroradiometer

^5^This study; Method: Spectra Vista Corporation (SVC) HR1024i spectroradiometer

^6^Schmidt et al., 2023; Method: ASD FieldSpec3 with contact probe

Some SWIR absorption features have been used to map polymer-bearing targets, including formal landfills, rooftops, and plastic debris aggregations on the surfaces of water bodies (Garaba et al., 2018; Biermann et al., 2020; Guo & Li, 2020; Hueni & Bertschi, 2020; Park et al., 2021; Zhou et al., 2021). AVIRIS imaging spectroscopy has been used to map plastic cover in a landfill and industrial area using features at 1215 and 1732 nm (Garaba & Dierssen, 2018). Spectral indices have also been developed, including the Floating Debris Index (Biermann et al., 2020), the Normalized Difference Plastic Index (NDPI) (Guo & Li, 2020), and other hydrocarbon indices (Asadzadeh & de Souza Filho, 2016). Plastic mapping algorithms based on SWIR reflectance alone can outperform algorithms using the entire spectrum (Schmidt et al., 2023).

Typically, polymer mapping accuracy is high for large plastic aggregations on dark, homogeneous backgrounds (i.e., ocean surface) (Biermann et al., 2020), and when using sub-decameter imaging spectroscopy data (Zhou et al., 2021). Accuracy is generally lower when applied to multispectral imagery in complex urban landscapes with diverse materials, high spectral heterogeneity, and aggregations of plastic or polymer-coated materials occurring at subpixel scales. Plastics have been classified in urban settings using WV3 SWIR imagery but subpixel-sized plastic aggregations were not mapped (Zhou et al., 2021). Estimating mixed quantities of several materials at the subpixel level may help map anthropogenic waste aggregations, which are irregularly shaped and include plastic mixed with other anthropogenic and natural materials.

For studies that compare laboratory-based spectra of plastic waste to satellite-based spectra of known or inferred plastic targets, measures of distance and similarity are complementary and have the best results when applied together (Carvalho Júnior et al., 2011). Spectral angle mapper (SAM) and spectral similarity value (SSV) are two approaches for comparing sample and reference spectra. SAM measures spectral similarity by comparing the spectral angle distance between a given spectrum and a reference spectrum (Gnann et al., 2022; Tasseron et al., 2022). SSV measures both the shape and distance similarity between two spectral classes by combining Euclidean distance similarity (EDS) and spectral correlation (*r*) (Thenkabail et al., 2007).

Sensors like Landsat and Sentinel-2 have limited applicability for mapping anthropogenic waste, since many urban targets are smaller than a decameter-scale pixel, and most plastic absorption features are not resolved by two broad SWIR bands (Schmidt et al., 2023). The WV3 SWIR sensor offers finer spatial (≈4 m pixels) and spectral (8 SWIR bands) resolution with substantial potential for urban plastic mapping (Livens et al., 2022). The most common urban plastics and many of their absorptions (Table 1) correspond to the location of WV3 SWIR bands 1–4 (Table S1).

When observed by Landsat (Sousa & Small, 2017) and Sentinel-2 (Sousa & Small, 2022; Small & Sousa, 2022), most terrestrial landscapes can be effectively described by a spectral feature space bounded by substrate (S), vegetation (V), and dark (D) endmembers. These endmembers along with potential plastic endmembers have yet to be fully characterized in WV3 SWIR imagery, and may constitute a significant component of image variance for some urban landscapes.

Matched filter (MF) models are distinct from spectral mixture models. MF models are designed to identify instances of a known target endmember. Originally developed for target detection in signal processing, MF uses a partial unmixing approach also known as constrained energy minimization (Harsanyi & Chang, 1994). MF models have been applied to spectral imaging and extended to include “mixture tuning” (Boardman, 1998; Boardman & Kruse, 2011). The goal of MF models is to estimate subpixel abundance while suppressing an unknown background in mixed pixels (Woodward, 1951; Turin, 1960) and thus may be particularly promising for mapping plastics.

### Research objectives and questions

More work is needed to fully characterize VSWIR absorption features for diverse urban plastics and to translate lab observations to standardize mapping methods with in situ observations of plastics in optically complex landscapes including rapidly developing urban areas. The objectives of this research are to (1) characterize the spectral diversity of polymers from discarded urban plastic waste located in dry stream channels in Los Laureles Canyon, Tijuana, Mexico, using laboratory reflectance spectroscopy; (2) quantify spectral separability between common polymers and other natural and human-made materials; (3) quantify spectral similarity between plastic waste and a WV3 image-derived endmember; (4) map cover of plastic and/or polymer-coated materials using an image endmember and MF algorithms applied to WV3 SWIR imagery; and (5) validate model performance with field observations and an accuracy assessment with visual interpretation of true color imagery. In pursuing these objectives, we answer the following research questions:

I. What SWIR wavelengths and WV3 SWIR wavebands are most useful in differentiating plastic or polymer-coated waste from other materials (i.e., vegetation, soil, other urban materials)? How do polymer absorptions differ (i.e., spectral location, depth) by polymer type?
II. When laboratory reflectance spectra are convolved to the WV3 spectral response function, what types of polymers are spectrally distinct from natural and other human-made materials and are more likely to be mapped with WV3 SWIR imagery of a heterogeneous urban environment?
III. How similar are WV3-convolved laboratory spectra of plastic and/or polymer-coated waste, vegetation, substrates, and other urban materials to WV3 image-derived spectra?
IV. With what precision can a matched filter map plastic and/or polymer-coated materials in WV3 SWIR imagery?

## Materials and methods

### Study area

**Fig. 1:**
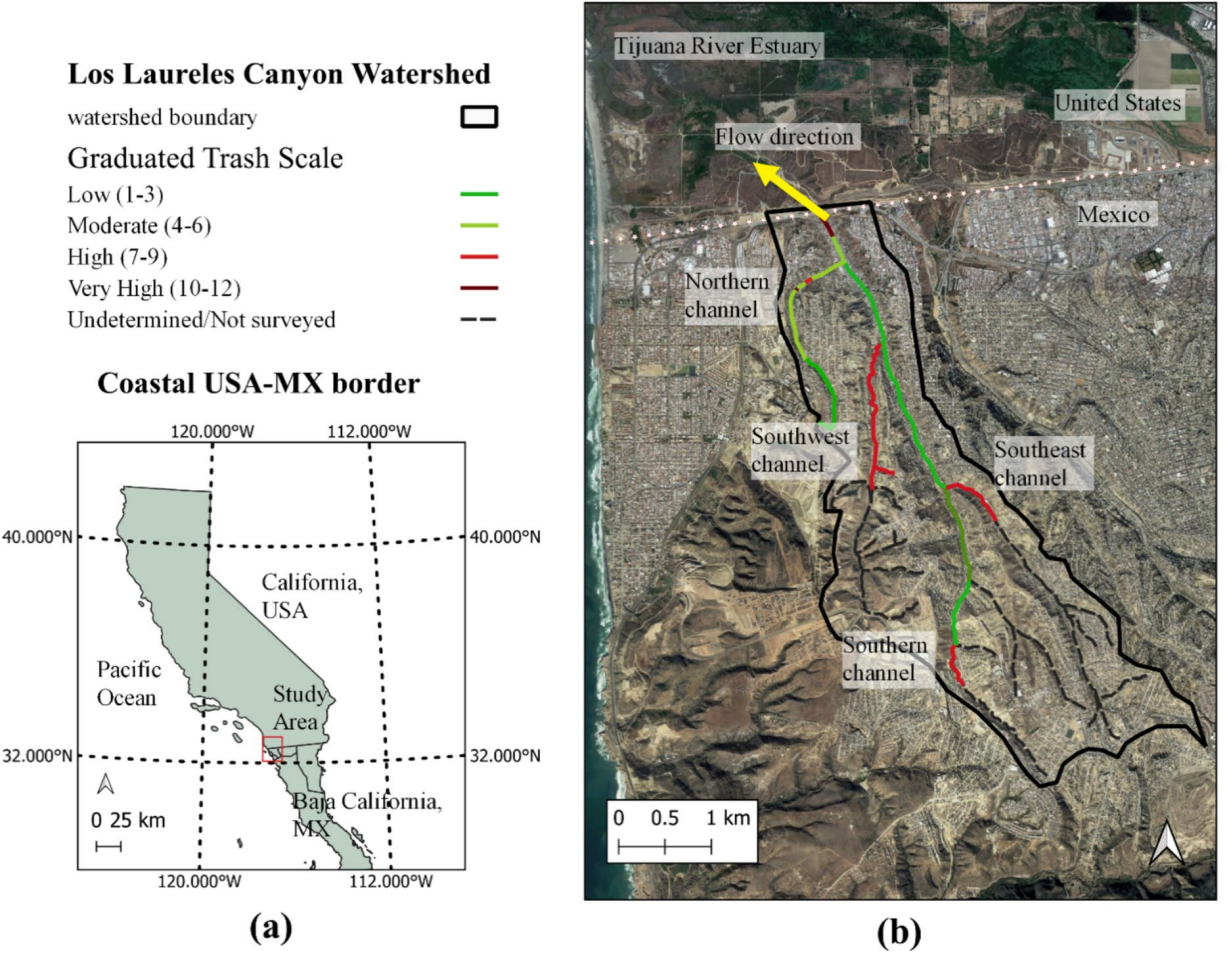
Index map of the Los Laureles Canyon Watershed (**a **red box indicates the study region; **b** thick black outline indicates watershed boundary) on the western end of the border between USA and Mexico. The entire study area shown in panel **b** is contained within a single WorldView-3 image. Map data: Google, ©2021 Landsat/Copernicus, Maxar Technologies

The Los Laureles Canyon Watershed, a 10.5-km^2^ coastal sub-basin in the Tijuana River Watershed, is located on the international border of San Diego, California, USA, and Tijuana, Baja California, Mexico (Fig. 1a). The watershed originates in Tijuana and discharges seasonally during storm events into the Tijuana River Estuary in California, USA (Fig. 1b). Los Laureles Canyon is characterized by numerous informal landfills, rapid population growth, and limited or intermittent solid waste collection, making it representative of many urbanizing watersheds globally. The main channel carries seasonal, unregulated cross-border flows that often include solid waste (Lopez-Galvez, 2014) and sediment (Gudino-Elizondo et al., 2019). Several communities in the watershed have high levels of socioeconomic marginalization (Biggs et al., 2014) and are impacted by plastic pollution that is exacerbated by erosion, floods, and landslides due to large rain events (Grover, 2011; Goodrich et al., 2020; Gudino-Elizondo et al., 2022). Large informal landfills often occur near unpaved, ephemeral stream channels and smaller waste aggregations are common throughout the watershed.

### Methodological overview

**Fig. 2:**
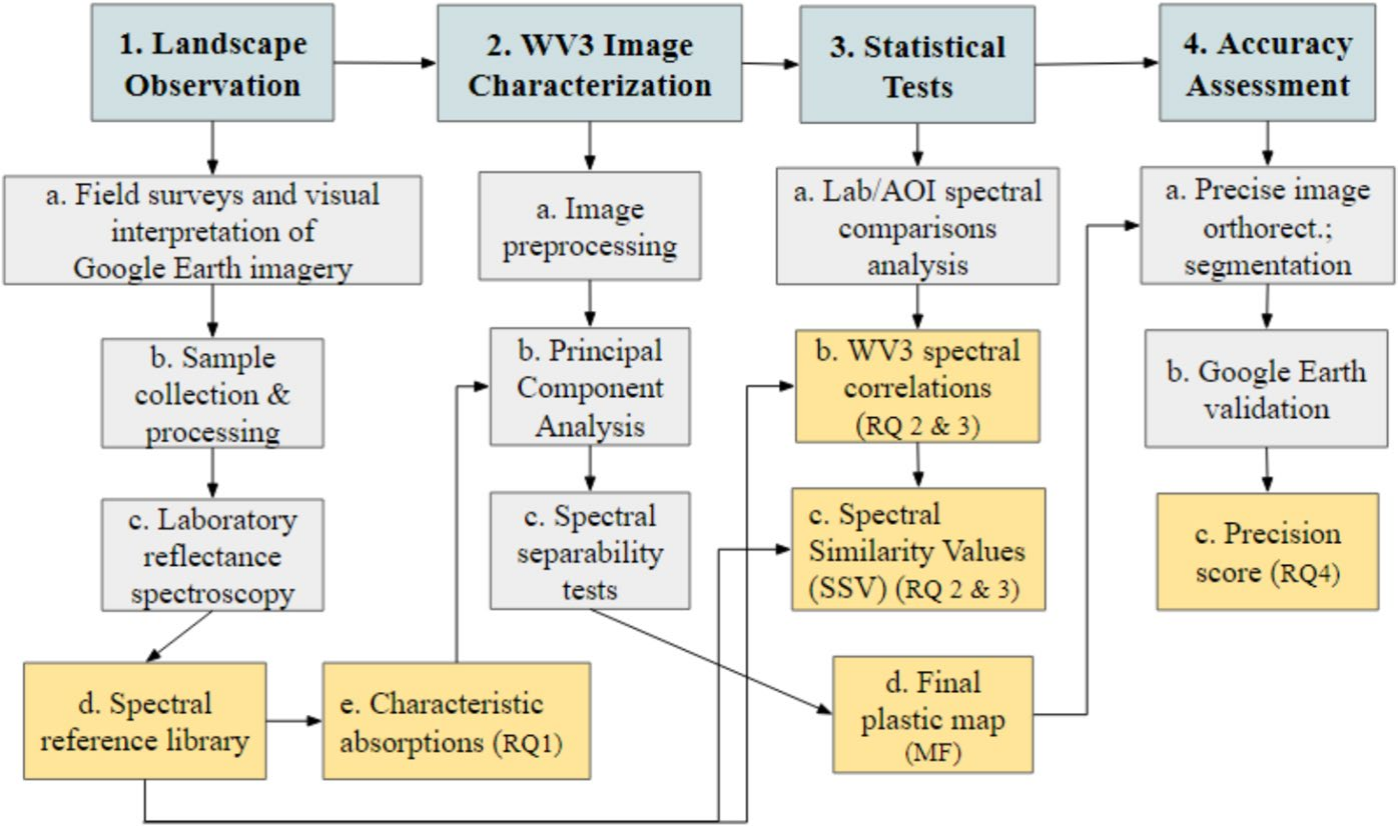
Study design and workflow. Broad stages in blue (top row); specific tasks in gray (middle), and outputs with corresponding research question (RQ) in yellow

Methods and workflow are summarized in Fig. 2. Briefly:

1. Abundance of trash in stream channels was surveyed and samples of plastic polymer-based or coated anthropogenic debris were collected during field visits;
2. Laboratory VSWIR reflectance spectra of the plastic samples were measured and convolved to the WV3 spectral response function;
3. WV3 SWIR image-derived endmembers were identified from spectral feature space and tested for spectral separability;
4. Spectra derived from the laboratory, image areas of interest (AOI), and image endmembers were compared using correlations and a spectral similarity test;
5. Maps of per-pixel plastic cover fraction were generated using matched filtering. Areas of spatially contiguous plastic and/or polymer-coated materials were identified and validated through visual evaluation of submeter true color imagery.

### Field surveys and sample collection

We conducted field surveys in October and November 2021 to identify and measure waste aggregations in stream channels. Field surveys assessed waste abundance levels in four channel reaches: northern, the southwest, southeast, and southern (Fig. 1). Reach-wise waste abundance was mapped following the Riverine Visual Observation method, which covers the channel plus a 5-m buffer onto the floodplain (Moore et al., 2020). Reaches were assigned one of four trash condition categories (TCC) of low, moderate, high and very high (Fig. 17 in Moore et al., 2020), and three numerical subcategories to further distinguish intra-category trash abundance levels: 1–3 (Low), 4–6 (Moderate), 7–9 (High), and 10–12 (Very High).

TCC values were recorded for 44 reaches and georeferenced using the Litterati smartphone application, including start and end locations, timestamp, and photos. Each observation included the TCC and numerical score, trash location, trash dispersal, and comments describing the main types of trash observed. Litterati data were later aggregated by stream reach.

**Fig. 3:**
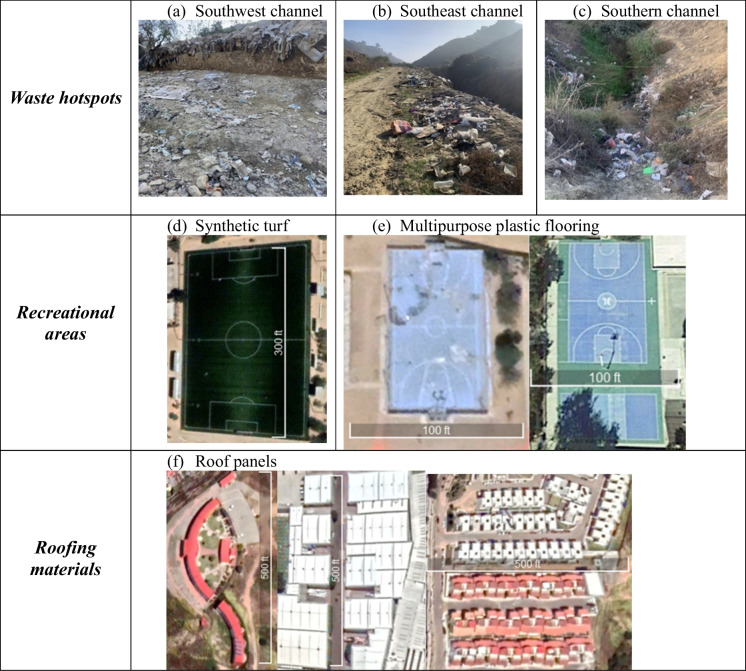
Examples of plastics and/or polymer-coated materials in the study area. Top row (**a**–**c**) shows example waste aggregations observed during 2021 fieldwork. Bottom rows (**d**–**f**) show nadir-looking true color aerial and satellite imagery examples of inferred plastic-bearing and/or polymer-coated infrastructure (source: Google Earth and Maxar)

Three reaches in the watershed had informal landfills with High or Very High TCC: the southwest, southeast, and southern (Fig. 1b). Waste aggregations varied in shape, size, and material composition. Household waste was often discarded away from the homes and into or along the stream channels, which are often steep and difficult to access. Unmanaged waste aggregations located along or within reaches were typically 4 to 30 m long. The largest observed dense (ground not visible) waste aggregations measured 80 to 150 m^2^ (Fig. 3a–c). Common plastic-bearing objects included household items and commercial product packaging. Common non-plastic objects included organic materials (e.g., food scraps) and construction materials (e.g., concrete, wood). The northernmost channel, in a more developed neighborhood, typically presented Low TTC scores (1–3), but suspected plastic-bearing and/or polymer-coated materials are also abundant in this area in the form of urban infrastructure (Fig. 3d–f), such as roof panels, synthetic turf fields, and multipurpose flooring in recreational areas. We inferred the presence of these materials due to consistent spectral signatures across these urban structures, resembling that of convolved plastic spectra (discussed further in the “Laboratory setup” section). These plastics and/or polymer-coated materials are not considered waste, and are not likely to be transported during storm events.

The areas that we mapped as having high quantities of waste coincide with observations by local non-governmental organizations that have worked in the watershed for over 10 years. This suggests that some aggregations and informal landfills may demonstrate geographic persistence despite dynamic changes in urbanization.

We collected 22 individual waste items from the southwest channel in December 2021. Object selection criteria included (1) collection feasibility (sufficiently compact to be held with one hand; larger materials were clipped), and (2) apparent visual diversity, including known or suspected differences in synthetic polymer type. Items spanned a wide range of colors, textures, translucence, and resin identification codes (RIC) (Aguilar, 2023). While diverse, the collection is not exhaustive of all plastic present in this complex urban landscape.

### Laboratory reflectance spectroscopy

Laboratory reflectance spectra were collected for each of the 22 waste items. In order to obtain a representative measurement of the plastic polymer itself, and to minimize capture of dirt or other coatings which accumulate in the field or in transport, items were gently cleaned in the laboratory. Some items heavily soiled with thick mud were rinsed with tap water, while others were wiped with a paper towel, and then all were air-dried overnight. The reflectance spectra of plastic samples coated in dirt is found to be similar to the reflectance spectra of clean plastic samples, so surface contamination of plastics should not impact the quality of spectral measurements (Masoumi et al., 2012). While some particulate matter still remained on some items, the overall purpose of sample cleaning was to establish reference measurements of relatively pure sample materials. These reference measurements establish a foundation for subsequent statistical and analytical investigation, including comparisons of spectral shape between laboratory spectra, image endmembers and other spectra of interest, and the final production of accurate MF maps.

#### Laboratory setup

Spectra were collected using a Spectra Vista Corporation (SVC) HR-1024i spectroradiometer, with NIST-traceable calibration to absolute radiance. The SVC spectroradiometer measures full-range (350–2500 nm) VSWIR reflectance with spectral resolutions of 3.3 nm at 700 nm, ≤ 9.5 nm at 1500 nm, and ≤ 6.5 nm at 2100 nm and nominal bandwidth is ≤ 1.5 nm between 350 and 1000 nm, ≤ 3.8 nm between 1000 and 1890 nm, and ≤ 2.5 nm between 1890 and 2500 nm. The SVC spectroradiometer mitigates spatial sampling complexities present in bundled foreoptic spectrometers (Mac Arthur, et al., 2012). Illumination simulating sunlight was provided by a 75 W tungsten-halogen Sunnex lamp powered by an Acopian A12H2100 power supply. Other lights in the laboratory room were shut off during the collection of measurements. A Spectralon® panel was used for white referencing to derive relative reflectance measurements from the SVC spectroradiometer. The spectroradiometer was connected to a handheld pistol mounted 16 in. above the measurement target area (Fig. S1). The tungsten-halogen Sunnex lamp was placed at a 60° angle and about 2 ft away from the waste items being measured. Spectra from the Spectralon® panel were first collected and then spectra of each macroplastic item over the panel. Waste items were oriented to maximize surface area. Each spectral measurement consisted of five continuous scans. Select items were measured at multiple orientations to observe any changes in spectra, which were deemed minimal after visual analysis.

#### Spectral library

Seventeen of the 22 waste items were plastic and included in the spectral library. Five items were not included because they were either non-plastic, of unknown polymer, or were black and spectrally featureless within the noise floor of the instrument (Aguilar, 2023; Table S1). The 17 plastic samples were categorized into polymer types using existing RIC or reference graphics from Tasseron et al. (2021) (Fig. 2 and Table 1) when RIC was not visible. An additional laboratory reflectance spectrum for PVC was obtained from the USGS Spectral Library, v7 (Kokaly et al., 2017). The spectral library for our study was limited to only these spectra in order to provide a representative sample of materials from our particular study area. The final spectral library included PET, HDPE, LDPE, PS and PVC, and a total of 18 plastic items (17 field samples and the USGS PVC item).

#### Convolution to the WorldView-3 spectral response function

**Fig. 4:**
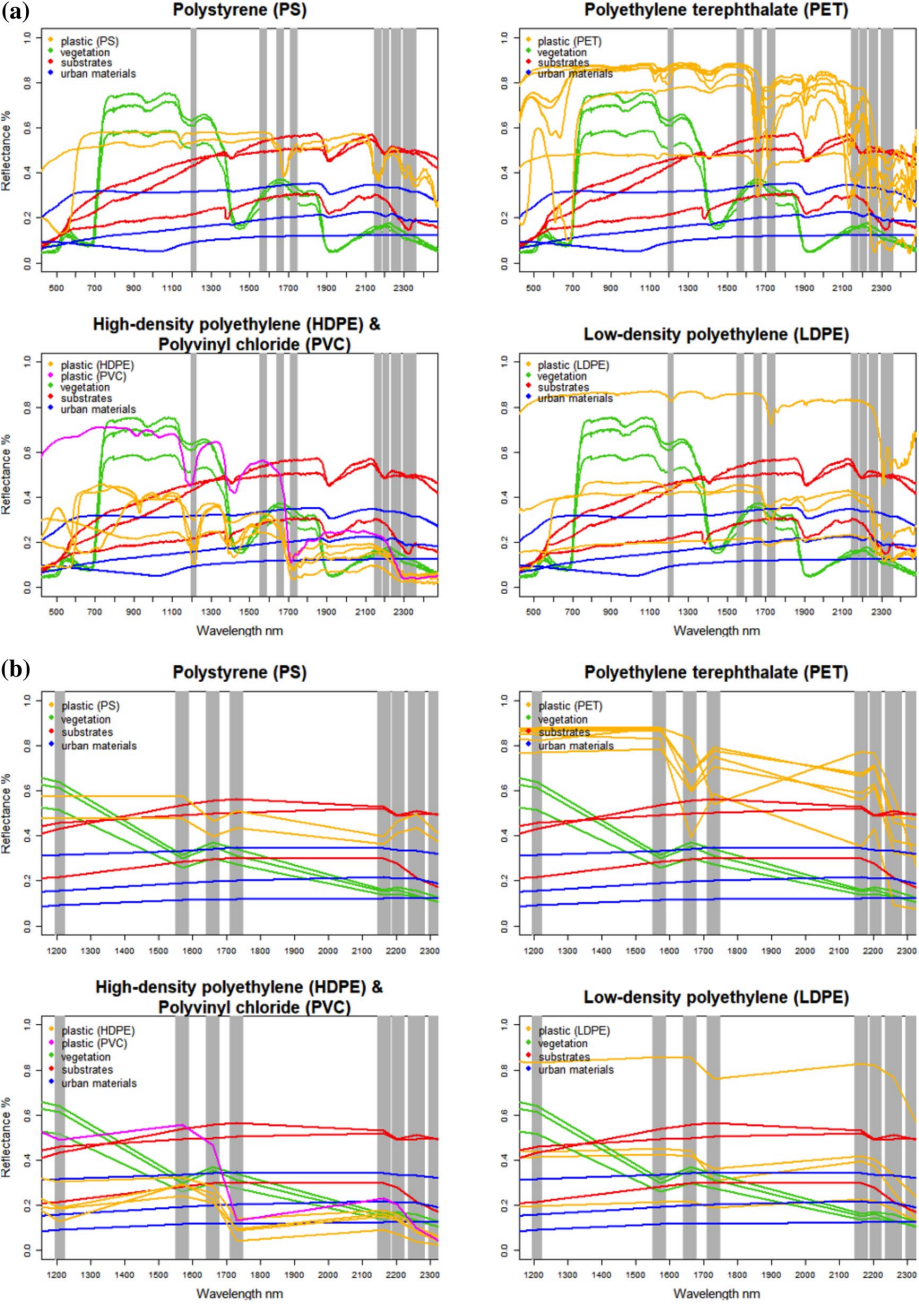
**a** Laboratory reflectance spectra of plastic waste items collected from our study area (yellow) are evaluated against Elvidge (1990) and Kokaly et al. (2017) laboratory spectra for common vegetation (green), substrates (red), and non-polymer anthropogenic materials (blue) (a–b). Locations of WV3 SWIR bandpasses are denoted by gray vertical bands. PS (Styrofoam, upper left;), PET (water bottles, upper right), HDPE and PVC (thick, cleaning product bottles, lower left) and LDPE (translucent mesh bags, lower right) (top four plots). **b** WorldView-3 spectra convolved from laboratory reflectance spectra (bottom four plots)

Figure 4 shows the laboratory reflectance spectra organized by polymer type, compared against the spectra of other materials from published spectral libraries of vegetation (Kokaly et al., 2017), natural substrates (Elvidge, 1990), and other non-plastic anthropogenic materials (Kokaly et al., 2017). Simulated multispectral WV3 SWIR spectra were computed by convolving the laboratory reflectance measurements with the published spectral response function of the WV3 sensor using ENVI software. Figure 4b shows these simulated WV3 spectra, for both the 18 plastic samples and the example spectra from other published libraries. Comparison of Fig. 4a and 4b reveals that some key plastic absorptions are retained by the eight WV3 SWIR bands, while others are lost. The greater retention of these absorption features is the physical basis for greater accuracy in plastic mapping using WV3 versus sensors with only two broad SWIR bands.

### Worldview-3 imagery

WV3 SWIR data of the study area were provided upon request through the NASA Commercial Smallsat Data Analysis (CSDA) program (Neigh et al., 2013) as National Imagery Transmission Format (NITF) Level-1B sensor corrected, unprojected (raw) data files. Acquisition dates for three cloud-free images were as follows: January 18, 2018; January 04, 2019; and May 03, 2020. Our study area was sufficiently compact to be entirely contained within a single WorldView-3 image. Atmospheric correction was explored via MODTRAN simulations using visibility data provided from the Tijuana Airport (Fig. S2). Rayleigh correction was implemented and found to have an impact on the order of 1%, as expected given the minimal impacts of a clear sky atmosphere on SWIR wavelengths. Final results were based on top-of-atmosphere reflectance (Chandrasekhar, 1960). We focused our analysis on the 2020 SWIR image since it was closest in time to the fieldwork, but all images were analyzed to assess the consistency of image endmembers. The 2020 image (Maxar ID: 104 A01005 A4B6 A00) was collected 27.5° off-nadir with a sun elevation of 69.2° and maximum target azimuth of 27.1°.

#### Image preprocessing

Digital numbers (DNs) of the WV3 Level-1B product were converted to top-of-atmosphere reflectance (ρ(TOA)λ) (Kuester, 2016) using R software. At-sensor radiance (L, Wμm^−1^ m^−2^ sr^−1^) was calculated from DN as:

1 $$L = GAIN * DN * (\frac{abscal\;factor}{effective\;bandwidth} ) + OFFSET$$

where GAIN and OFFSET are absolute radiometric calibration adjustment factors published by the sensor operator, effective bandwidth is the relative spectral radiance response for each band delivered in the image metadata, abscal factor is an absolute calibration factor also delivered in image metadata, and DN is the digital number of each pixel in the image.

From at-sensor radiance, exoatmospheric reflectance was then calculated for each pixel at a given wavelength:

2 $$\rho {\left(TOA\right)}_{\lambda } =\frac{{L}_{\lambda }{d}^{2}\pi }{{E}_{\lambda }cos{\theta }_{s}}$$

where *L*_λ_ is the at-sensor radiance computed in Eq. 1, *d*^2^ is the two-way Earth and Sun distance in astronomical units, *E*λ corresponds to the Thuillier (2003) solar exoatmospheric spectral irradiance values (W m^−2^ μm^−1^) averaged by band (Kuester, 2016; Table 2), and θ_s_ is the solar zenith angle at the time of image acquisition.

#### Principal component analysis and image endmembers

**Fig. 5:**
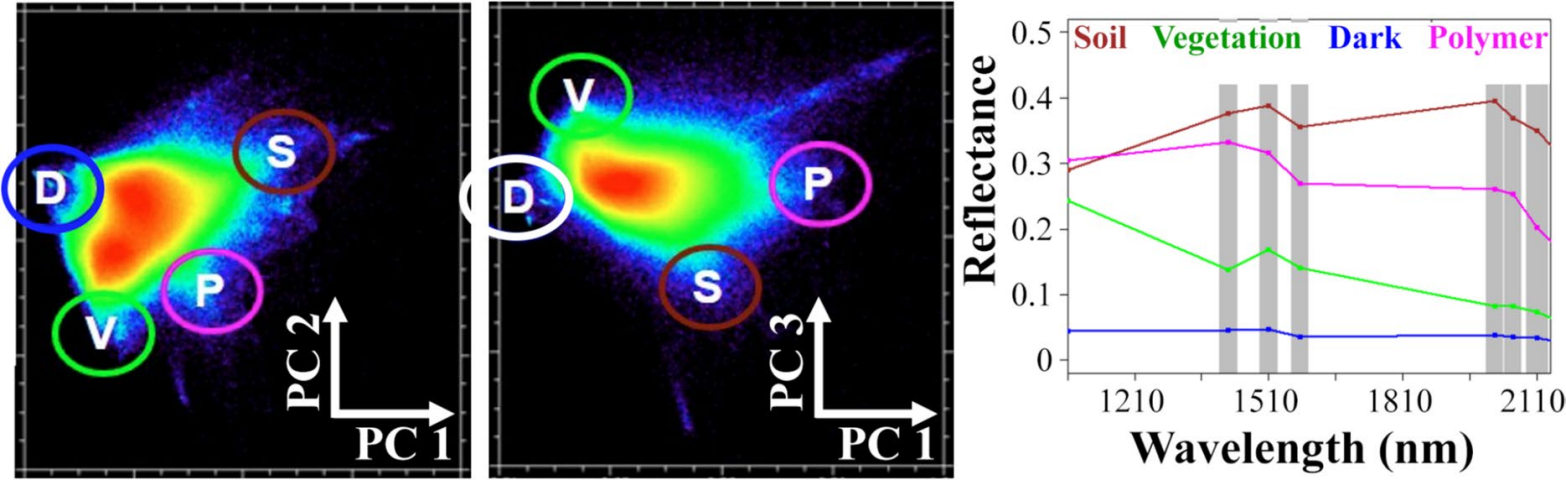
WorldView-3 SWIR spectral feature space. Image-derived endmember reflectance spectra (right) are multipixel means (*N* ≅ 8000 pixels) from four circled apexes of the low-order feature space (left). Endmembers correspond to dry, SWIR-bright targets like soils (S), vegetation (V), SWIR-dark targets and shadows (D), and polymers (P)

Image-based endmembers were selected by investigation of the low-order spectral feature space. A covariance-based principal component (PC) transformation was applied to the WV3 SWIR image. Image-based endmembers (Fig. 5) were manually selected from bounding apexes of the point cloud, corresponding to high albedo substrates (S), green vegetation (V), shadow and dark (D) materials, and polymers (P). The image-derived plastic endmember spectrum (Fig. 5, magenta) exhibits a spectral signature similar to most of the WV3 simulated plastic spectra (Fig. 4b, yellow) with features observed at 1570–1750 nm (bands 2–4) and 2145–2285 nm (bands 5–7).

Spectral separability of the image-derived endmembers was quantified using Jeffries-Matusita and Transformed Divergence metrics (Jeffreys, 1997; Richards & Jia, 1999), which range from 0 (complete overlap) to 2.0 (high separability). Image endmembers (SVDP) had high separability (2.0) between all pairwise combinations (Table 2).

**Table 2:** Spectral separability of endmember ROIs using the Jeffries-Matusita (J-M) and Transformed Divergence (TD) metrics

Separability (both J-M and TD)	Vegetation	Substrate	Dark	Polymer
Vegetation	0	2.0	2.0	2.0
Substrate	2.0	0	2.0	2.0
Dark	2.0	2.0	0	2.0
Plastic	2.0	2.0	2.0	2.0

### Statistical tests

#### Correlation tests: laboratory spectra

Pearson correlation coefficients were computed between the WV3-convolved lab spectra for different polymer samples and other materials. Spectral correlations quantify the similarity or dissimilarity of spectral shape and curvature without regard to amplitude. Two waste items were selected to represent each of the five polymer types, except for PVC, where only one measurement was available.

Correlation coefficients were calculated between the laboratory spectra and library spectra of vegetation, natural substrates (soil, gravel, and sandstone), and anthropogenic materials (asphalt, concrete, metal). We also computed a mean laboratory plastic spectrum (MLPS) for the 18 plastic sample spectra in our library. The MLPS was generated from 4 spectra of HDPE, 4 spectra of LDPE, 7 spectra of PET, 2 spectra of PS, and 1 spectrum of PVC. The image-derived plastic endmember spectrum was tested for correlation with the convolved spectra of nine selected plastic samples from the spectral library and the convolved MLPS. All lab data processing, statistics, and plots were generated in R software.

#### Area of interest–derived spectral profiles

We hand-digitized areas of interest (AOIs) of homogenous land cover types using visual interpretation of both pansharpened WV3 VNIR imagery (coincident with SWIR image) and high-resolution Google Earth imagery (collected 9 days after the May 2020 WV3 image acquisition), and calculated mean WV3 reflectance spectra for each AOI. Vegetation and natural AOIs included a small grove of riparian trees, grass, a shrub-covered hillside, and a bare dirt lot. Urban substrate AOIs included an empty asphalt parking lot and concrete. Polymer AOIs included a waste patch (aggregation) in the southeast channel, a recreational court, and white and red rooftops. For the waste patch AOI, we located large waste areas in the imagery and corroborated these against independent fieldwork. Through early conversations with collaborators with extensive field experience in the watershed, we learned the largest informal landfills in the study area typically exist on the timescale of months to years. All AOIs were then overlaid on the WV3 image and clipped to compute mean spectral reflectance for each polygon. AOI sizes varied between 16 (waste aggregation) and 222 (shrub hillside) pixels. Pearson correlation coefficients were calculated between the AOI spectra and the WV3-convolved lab spectra. These comparisons provide additional validation that the convolved laboratory spectra coincide with materials in the image, and evaluate the extent to which materials are spectrally distinct in WV3 SWIR.

#### Spectral similarity value

The spectral similarity value (SSV) is defined as (Thenkabail et al. 2007):

3 $$SSV= \sqrt{{EDS}^{2}+(1-\rho {)}^{2}}$$

where EDS is the normalized Euclidean distance similarity between two class spectra and ρ is the square of the correlation coefficient between the two spectra (the “Correlation tests: laboratory spectra” section). Historical minimum–maximum normalization maintains consistency and comparability across time and different instruments and environmental conditions. We normalized EDS to 0 to 1 as:

4 $${EDS}_{normal}=\frac{D-\text{min}(D)}{\text{max }\left(D\right)-\text{min}(D)}$$

where EDS is the Euclidean distance between two spectra, and *D* is the vector of the *D* values for all pairwise comparisons between image- (AOI) and laboratory-derived spectral measurements. SSV scores range from 0 (most similar) to 1.414 (most dissimilar).

### Matched filtering

We used matched filtering (MF) (Turin, 1960; Boardman, 1998) to compute the strength of relationship between the targeted reflectance spectrum of each pixel in the image and the plastic endmember spectrum. Matched filters maximize the response of a known endmember and suppress the response of a composite unknown background. Pixels with high similarity to the plastic endmember spectra receive high scores, with values ranging from 0 to 1.

#### Image coregistration and segmentation

Large (20–30 m, 4–8 pixels) geolocation and orthorectification errors were evident in WV3 satellite imagery, likely exacerbated by the topography. To improve coregistration, we exported an ArcGIS Google Earth basemap at 0.5-m pixel resolution and coregistered the WV3 SWIR and pansharpened VNIR images to this basemap using AROSICS (Scheffler et al., 2017, https://github.com/GFZ/arosics). AROSICS performs automated image-to-image co-registration using both global and local cross correlation. We obtained satisfactory results using individual coregistration routines on the WV3 image subset to northern and southern halves of the watershed, including a window size of 400 × 400 pixels, maximum 10 iterations, maximum shift of 60, and tie point grid pixel spacing of 60 (Refer to Figure S1 in Aguilar, 2023 for an example of the correction). Final remaining maximum geolocation discrepancies were visually evaluated at 1–2 pixels (4–10 m) in the most severe locations, smaller than the AOIs used for validation (150–450 m^2^).

#### Accuracy assessment

**Fig. 6:**
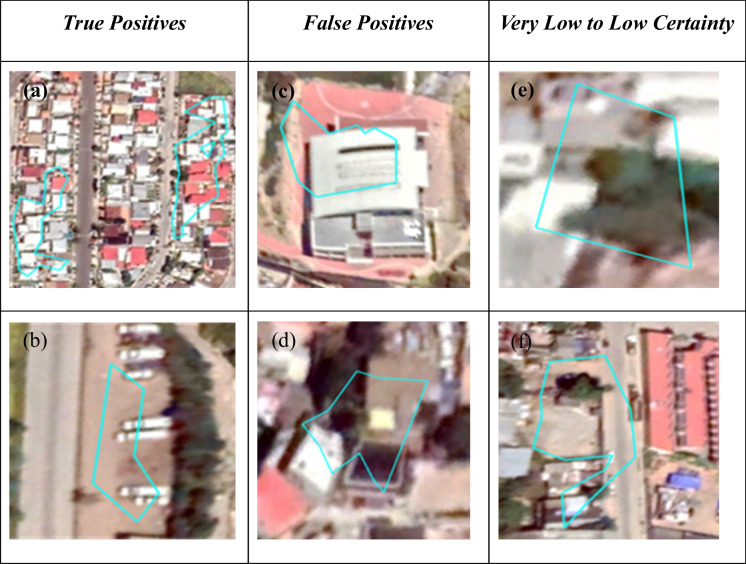
Examples of the validation process. True Positives (**a**–**b**): Mixed red and white roofs, either comprised of polymer building materials or coatings (**a**) = Certainty Level: 5; Stadium roof and multipurpose flooring comprised of polymers, or with polymer coatings (**b**) = Certainty Level: 4. False Positives (**c**–**d**): Trucks and dirt lot (**c**) = Certainty Level: 5. Trees and roofs (**d**) = Certainty Level: 4. Very Low to Low Certainty Scores (1–2; **e**–**f**): Unclear structures/objects (**e**) = Certainty Level: 1. Poor registration of roof (**f**) = Certainty Level: 2

Clusters of spatially contiguous pixels with inferred plastic MF scores in the top 20 th percentile (MF > 0.37) were identified from the output raster, with a minimum size of 20 pixels. Clusters were then vectorized, resulting in > 1000 individual polygons. Two hundred of these polygons were randomly sampled, with areas ranging from 150 and 450 m^2^ and then validated against high resolution true color imagery. Polygons were examined visually and labeled as either inferred plastic (P) or non-plastic (NP) and assigned a Level of Certainty score (1–5). Samples with certainty scores 1 and 2 were defined to have Very Low to Low Certainty, respectively. This typically included areas with poor geolocation/orthorectification and/or unclear structures and materials (Fig. 6). Scores of 3 were grouped with Low Certainty classifications, and scores of 4 and 5 were considered High to Very High Certainty, respectively. High and Very High Certainty included polygons that overlaid areas and objects clearly, and where the material could be unambiguously identified as polymer or non-polymer (Fig. 6). Accuracy was assessed using both Google Earth imagery and pansharpened WorldView-3 VNIR imagery.

True positives (TP) and false positives (FP) were then assigned to each polygon based on the visual categorization. Precision scores were calculated as the ratio of TP to the sum of total predictions:

5 $$Precision=\frac{TP}{TP+ FP}$$

This analysis quantifies commission errors (Type I) and model precision. We did not compare the reach-wise trash abundance scores from our field timelines to the satellite classification due to the time difference between survey and WV3 image acquisition. Instead, we used knowledge from the field to help find unmanaged waste in the May 2020 image, since large waste aggregations are typically persistent. Errors of omission (Type II) were not evaluated due to the absence of independent, field-based measures of plastic abundance for our study area from May 2020. Our evaluation of potential Type II errors thus relies on statistical analysis of spectral separability using information from reflectance spectroscopy and the imagery. Correlation coefficients and SSV scores between sample spectra allowed us to further interpret the accuracy assessment and address mapping limitations.

## Results and discussion

### SWIR-differentiated plastics

#### Characteristic absorptions and key WorldView-3 SWIR wavebands

The 17 laboratory-measured reflectance spectra for PET, LDPE, HDPE, and PS (plus 1 previously published PVC spectrum) (18 total) have several consistent absorptions in the shortwave infrared (SWIR) wavelengths occurring near 1210, 1415, 1660, and 1728 nm, and smaller, consecutive absorptions between 2132 and 2313 nm (Fig. 4a). These characteristic absorptions were also apparent in the MLPS (Fig. S3). All polymer types exhibited a steep drop in reflectance beginning at 2200 nm (Fig. 4a; Fig. S3). Simulated WV3 spectra with retained features near 1210 nm were present in HDPE and PVC and overlapped with SWIR waveband 1; features near 1570–1730 nm were present in all five polymers and overlapped with SWIR wavebands 2 to 4, and features near 2100–2300 nm were present in PET, PVC and LDPE, and overlapped with SWIR wavebands 5 to 8 (Fig. 4b).

#### Differences in absorptions by polymer type

Band centers and visually assessed absorption depths depended on polymer type. PS absorptions differed most from other types, with prominent features at 1678 and 2167 nm and a minor absorption at 1141 nm (Fig. 4a). The resampled spectra for PS retained absorptions within WV3 SWIR waveband 3. HDPE, LDPE, and PVC showed consistent absorptions between 1206–1213 nm, 1400–1430 nm, and 1728 nm, though absorption depth was always greater in HDPE and PVC (Fig. 4a). PET had consistent absorptions at 1415, 1660, 1908, and 2132 nm (Fig. 4a). PET and LDPE demonstrated the steepest downward trend in the longest SWIR wavelengths (Fig. 4a), which was clearly retained in the simulated WV3 spectra of PET (Fig. 4b).

### Spectral comparisons: plastic versus other materials

WV3-convolved spectra of vegetation, substrates, and other common non-plastic urban materials were not highly correlated to the WV3 convolved spectra of the plastic polymer samples (Table 3a) and had high SSV scores (Table 3b), suggesting good spectral separability and minimal confusion between spectrally distinct plastics and other materials. The image polymer endmember correlated most closely (*p* < 0.01) and was most similar (low SSV) to the lab spectra of HDPE, LDPE, and PVC and the MLPS (Table 3a–b). The image polymer endmember also looks similar to spectra of PET, HDPE, LDPE, PVC, and the MLPS (Fig. S4). The two PS objects had different absorption features than the rest of the waste items and also the weakest correlations and highest SSV with the image polymer endmember (Table 3a–b). The PET spectrum correlated closely with the image polymer endmember (0.88–0.89, *p* < 0.01). Despite PET being the most abundant polymer type used to generate the MLPS, the SSV was relatively high (> 0.4; Table 3b) due to general distance in spectral reflectance. The MLPS and four plastic lab spectra (one PET sample, one LDPE sample, one PS sample, and PVC) correlated with the vegetation lab spectrum (*p* < 0.05) (Table 3a), but the SSV indicated good separation between vegetation and the plastic spectra (Table 3b). SSV was consistently below 0.4 between the image endmember and the laboratory spectra (except PS and PET) and above 0.4 for all non-plastic materials, so SSV values lower than 0.4 are highlighted in bold in Table 3b.

**Table 3:** Pearson correlation coefficients (**a**) and spectral similarity values (SSVs) (**b**) between laboratory-measured spectra of field-collected plastics, a WV3 image endmember, the mean laboratory plastic spectrum (MLPS), and other materials of interest. Other laboratory spectra are from Elvidge (1990) and Kokaly et al. (2017)

A. Laboratory spectra versus published spectral libraries: Pearson correlation coefficients											
Convolved SWIR correlations 1209–2330 nm *p* < 0.01** | *p* < 0.05*		PLASTIC LABORATORY SPECTRA									USGS 2017
		MLPS *18 items	PET		HDPE		LDPE		PS		PVC
			Item 1	Item 2	Item 1	Item 2	Item 1	Item 2	Item 1	Item 2	Item 1
MLPS *18 items		–––	0.85**	0.87**	0.94**	0.89**	1.00**	0.91**	0.62	0.73*	0.99**
WV3	Plastic endmember	0.95**	0.88**	0.89**	0.88**	0.83**	0.94**	0.95**	0.55	0.7	0.90**
OTHER LAB SPECTRA	Vegetation	0.73*	0.76*	0.49	0.49	0.42	0.75*	0.59	0.67	0.71*	0.71*
	Sand	− 0.03	− 0.03	0.04	0.17	0.25	− 0.03	0.09	− 0.23	− 0.09	− 0.05
	Soil	− 0.39	− 0.4	− 0.17	− 0.18	− 0.12	− 0.4	− 0.18	− 0.68	− 0.59	− 0.41
	Gravel	0.45	0.51	0.64	0.48	0.44	0.42	0.68	0.03	0.2	− 0.35
	Asphalt	− 0.43	− 0.45	− 0.04	− 0.23	− 0.21	− 0.45	− 0.12	− 0.56	− 0.51	− 0.47
	Concrete	0.01	0.08	0.27	0.12	0.12	− 0.01	0.29	− 0.22	− 0.1	− 0.06
	Metal	− 0.66	− 0.68	− 0.43	− 0.4	− 0.31	− 0.67	− 1.5	− 0.66	− 0.65	− 0.65
B. Laboratory spectra versus published spectral libraries: spectral similarity values; lower values indicate higher similarity; values less than 0.4 are in bold											
Spectral similarity values (SSV) (0–1.414)		PLASTIC LABORATORY SPECTRA									USGS 2017
		MLPS *18 items	PET		HDPE		LDPE		PS		PVC
			Item 1	Item 2	Item 1	Item 2	Item 1	Item 2	Item 1	Item 2	Item 1
MLPS *18 items		–––	0.5	0.58	**0.3**	0.41	**0.19**	**0.16**	0.64	0.5	**0.06**
WV3	Plastic endmember	**0.16**	0.65	0.74	**0.24**	**0.37**	**0.13**	**0.13**	0.72	0.58	**0.24**
OTHER LAB SPECTRA	Vegetation	0.49	0.72	1.06	0.78	0.87	0.46	0.68	0.61	0.59	0.52
	Sand	1.04	1.05	1.06	1.13	1.14	1.12	1.02	0.95	0.99	1.1
	Soil	0.89	0.91	1.05	1.11	1.16	0.97	0.99	0.54	0.65	0.94
	Gravel	0.82	0.98	0.95	0.78	0.83	0.83	0.55	1.02	1.00	0.9
	Asphalt	0.86	1.1	1.32	0.95	0.96	0.8	1.01	0.76	0.84	0.83
	Concrete	1.01	1.13	1.12	1.02	1.04	1.02	0.92	0.95	1.00	1.03
	Metal	0.7	1.04	1.29	0.84	0.9	0.56	0.84	0.73	0.79	0.68

### Spectral comparisons: laboratory versus image AOI

The mean reflectance spectra of the three independently derived AOIs with suspected plastic-bearing and/or polymer-coated materials (one waste aggregation, one recreational court, and one area with white and red rooftops) correlated most closely to the MLPS (Table 4a; *r* = 0.94, 0.98, 0.97, respectively). The MLPS also correlated with the vegetation spectrum (Table 4a; *r* = 0.71, 0.85, 0.84), but the SSV indicated slightly greater separation (Table 4b, green box). The MLPS had the lowest (most similar) SSV when compared to the spectra from plastic AOIs: a waste patch (0.15), the white and red roofs average (0.06), and an outdoor court (0.22) (Table 4b, magenta box). The SSVs between vegetation, substrates, and non-plastic urban materials and the plastic AOIs were high, indicating low similarity and good separability, except for a low SSV (0.39) between gravel and the recreational court AOI (Table 4b).

**Table 4:**
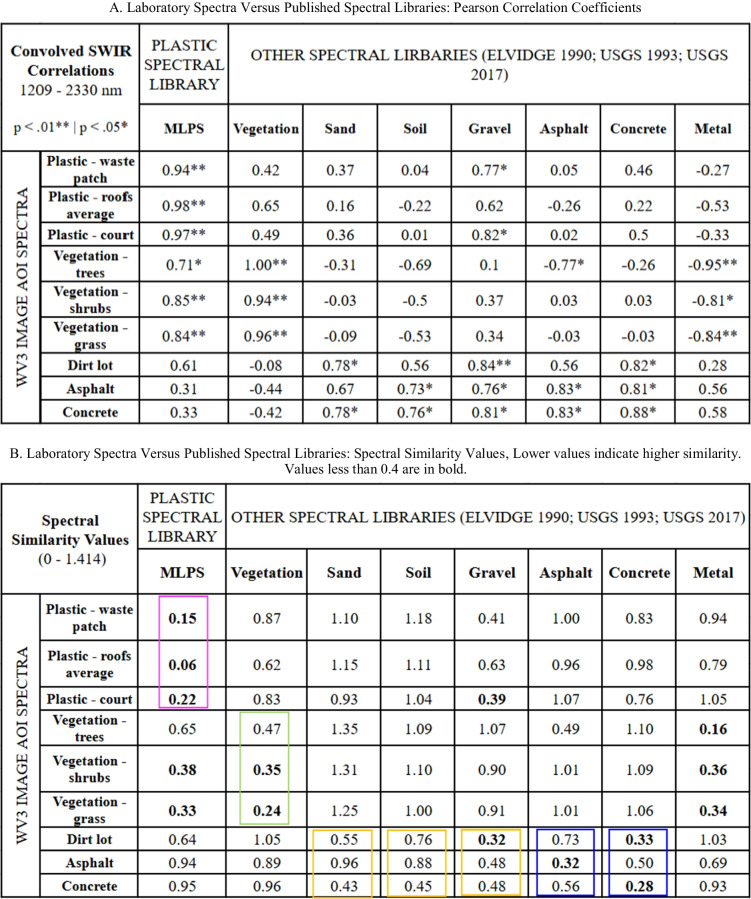
Pearson correlation coefficients (**a**) and spectral similarity values (**b**) between laboratory-measured spectra and image-derived spectra from areas of interest (AOIs). In Table 4b, the magenta box represents plastic AOI-lab (A-L) pairs, the green box represents vegetation A-L pairs, the orange box represents substrate A-L pairs, and the blue box represents other potential non-plastic urban material A-L pairs

### Matched filtering

**Fig. 7:**
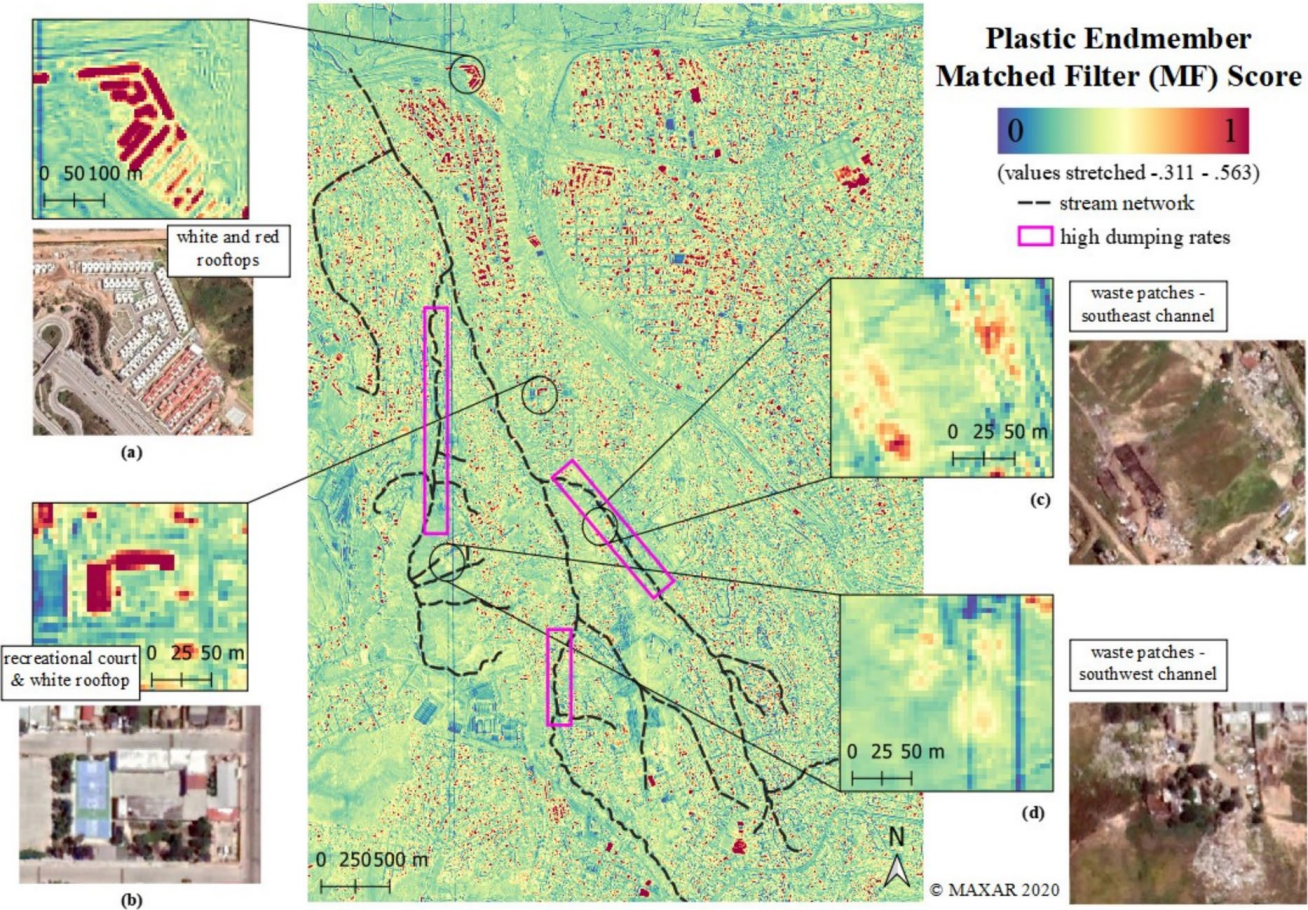
Matched filter results (© Maxar 2020), and reference imagery from April 24, 2020 (© Google Earth and Maxar) for each circled location. Magenta boxes outline general channel areas with informal landfills. Plastic-bearing and/or polymer-coated urban infrastructure, such as red and white rooftops (**a**) and recreational courts (**b**) have the highest MF scores

The largest spatially contiguous clusters identified by the MF contain residential, commercial, and industrial roofing materials, likely to be either comprised of plastic-bearing materials or coated with polymer-based paints (Fig. 7). Suspected plastic cover is high across the northern, more developed portion of the watershed (Fig. 7). MF identified two of the three field-documented waste aggregations in the southwest and southeast channel (Fig. 7), but did not identify appreciable plastic cover in the southernmost channel, though large waste aggregations were observed during field visits. This suggests potential undocumented trash deposition and removal between the image acquisition (2020) and field observations (2021). Areas with high MF scores also included some recreational courts and track fields, which can be made from various plastic materials and are often coated with polymer-based paints. The unmixed image had low MF scores for a synthetic turf field (Fig. 3d) and two other known synthetic turf fields, likely due to very low reflectance values across all SWIR wavelengths (< 0.09). The largest waste patches (aggregations) observed in the southwest (c) and southeast channels (d) have lower MF scores and through Google Earth resemble patchy discolorations on natural terrain usually in open canyon areas upslope of the channels.

#### Precision and level of certainty

Of the 200 polygons assessed, 185 were true positives (TP) and 15 were false positives (FP) for plastic-bearing and/or polymer-coated materials, resulting in a precision score of 92.5% (Table 5). Rooftops in residential, commercial, or industrial areas were identified in at least 180 of the 200 polygons. Objects in the polygons were categorized as roof types, either “White,” “Red,” “Mixed” (combination of red and white roofs), and a separate category, “Other” (Table 6). The Other category includes some misclassifications of trees near other structures (e.g., parking lots, roads), as well as plastic-bearing and/or polymer-coated roofs and areas with more specific descriptions (e.g., sports stadium, waste patch, greenhouses).

**Table 5:** Precision scores. Sample polygon counts for each classification outcome and certainty level are presented

Certainty level (CL)	True positives (plastic)	False positives (not plastic)
1 (Very Low)	2	1
2 (Low)	14	2
3 (Neutral)	8	4
4 (High)	22	4
5 (Very High)	139	4
Total	185	15
Samples	Counts	Precision %
All CLs	185/200	92.5
CLs 4–5	161/169	95.3

**Table 6:** Level of certainty scores or the 200 model-classified polygons. Mixed = red + white roofs

Certainty level (CL)	Polygon counts	% of total polygons	White roofs	Red roofs	Mixed roofs	Other
1 (Very Low)	3	1.5	0	0	2	1
2 (Low)	16	8.0	5	1	8	2
3 (Neutral)	12	6.0	1	2	4	5
4 (High)	26	13.0	5	2	14	5
5 (Very High)	143	71.5	48	17	71	7
**Total**	**200**	**100%**	**59**	**22**	**99**	**20**

If the accuracy assessment is restricted to polygons with High to Very High Certainty (4–5), the number of true positives becomes 161 out of 169 polygons and a precision score of 95.3% (Table 5).

### Discussion

#### Laboratory spectroscopy

The laboratory spectroscopic results of terrestrial macroplastic waste generally agree with other studies (Garaba & Dierssen, 2018; Moshtaghi et al., 2021; Tasseron et al., 2021; Knaeps et al., 2021; Schmidt et al., 2023), which find consistent absorptions across all polymer types and absorptions that depend on polymer type. For PET, HDPE, LDPE, PS, and PVC, absorptions features were consistently observed around 1210, 1415, 1660, and 1728 and between 2132 and 2313 nm (Fig. 4a). The first four wavelengths coincided closely with previous work (Garaba & Dierssen, 2018; Moshtaghi et al., 2021; Tasseron et al., 2021; Knaeps et al., 2021; Schmidt et al., 2023). In our samples, absorptions at 1908 nm were unique to PET samples and absorptions at 1141, 1678, and 2167 nm were unique to PVC. Absorptions at 1210 nm (SWIR-1) and 1415 nm may not be useful for mapping plastic due to potential confounding spectral similarity to the vegetation spectra used in this analysis and due to atmospheric absorptions (Garaba & Dierssen, 2018). Minor absorptions at the longest wavelengths (2132–2313 nm) have not been consistently identified in previous studies, with the exception of 2046 nm and 2313 nm (Garaba & Dierssen, 2020).

Our laboratory spectroscopy results suggest that plastic absorptions in the longer wavelengths, beyond 1660 nm, may be more characteristic of plastic and facilitate detection using bands SWIR-3 and higher. Polymer spectra convolved to the WV3 SWIR response function retained features near 1210 nm for HDPE and PVC; 1570–1730 nm for PS, PET, HDPE, PVC, and LDPE; and 2100–2300 nm for PET, PVC, and LDPE. Due to these features, our final map was generated using all SWIR bands. Spectra generally showed decreasing reflectance with increasing wavelength for all polymer types but the decrease was steepest in PET, HDPE, and PVC. Downward spectral slopes in these wavelength regimes have been previously linked to plastic in urban areas (Garaba & Dierssen, 2018; Guo & Li, 2020).

#### Image analysis

The combination of WV3 SWIR urban feature space characterization, with independent corroboration from reflectance spectroscopy, is a contribution of this study to watershed-scale plastic mapping in complex urban environments. Our plastic image endmember spectrum correlates with the WV3-convolved spectra of common urban plastics like HDPE, LDPE, PVC, and PET. These spectra show general decreasing reflectance characteristics between 1550–1750 nm (SWIR-2 to SWIR-4) and 2145–2285 nm (SWIR-5 to SWIR-7). Our plastic image endmember spectrum also resembles WV3-resampled field spectra (Guo & Li, 2020), resampled laboratory spectra of aliphatic plastics (Zhou et al., 2021), and absorptions found in PE (Heiden et al., 2007). Several WV3 SWIR bands, 2–4 and bands 5 and beyond, may be important for differentiating plastic polymers from other broad land cover classes and highlight the utility of multiband SWIR sensors like WV3 in plastic mapping.

Correlations among plastic spectra derived from the laboratory and image AOIs are generally higher than those between plastics and non-plastic materials. In some cases, laboratory spectra for plastic also correlated with spectra from vegetation. This may be related to compositional similarity at the molecular level associated with the abundance of structural hydrocarbons in each material. SSV between vegetation and plastic spectra were relatively higher, indicating larger Euclidean distance.

Our correlation coefficients and SSV scores between the laboratory spectra and the spectra of select AOIs suggest that suspected plastic-bearing and/or polymer-coated targets can be discriminated from common non-polymer materials in our study area. The low SSV between the MLPS and the image area of interest (AOI) for plastic roofs suggests that many of the white and red roofs in the study area may be of or coated with polycarbonates or plastics with aliphatic compounds such as PVC, PE, or a polymer with similar spectral features. Our results are comparable to the detection of synthetic hydrocarbons in industrial roofs using spectral features of marine-harvested macroplastics with prominent features at 1215 nm and 1732 nm (Garaba & Dierssen, 2018). Other areas with high density of plastic materials such as waste aggregations and outdoor courts also had the lowest SSV scores compared to all other AOI-lab comparisons between vegetation, substrates, and other urban materials.

#### Image endmember maps

Our results affirm the potential for sub-decameter multispectral SWIR satellite imagery to assist in mapping and monitoring anthropogenic debris in complex urban areas. Our approach detected different types of urban plastic in our study area; our maps are of suspected plastic-bearing materials based on a WV3 image endmember that looks like, correlates with, and has spectral similarity with laboratory spectra of plastic samples.

More organized efforts are needed to obtain field data for this study area so a full validation can be performed. The generalizability of the spectra of these materials also remains unknown. Considerable geographic variability may exist in the relative and absolute abundances of different plastic polymer types, including some polymers that were not collected during our fieldwork. Future work could include in-depth analysis of reflectance spectra from a much broader, more globally representative range of plastic types, weathering conditions, and sources (e.g., agricultural plastic mulches).

#### Limitations

Our approach detects two very different types of targets: (1) objects constructed from plastic materials, and (2) objects constructed from non-plastic materials but coated in polymer-based paints. This situation, a manifestation of the fundamental problem of non-uniqueness in remote sensing, can be observed via the prominent detection of roofing materials by the matched filter. Some of these roofing materials may indeed be comprised of plastic sheeting and/or skylights, but other materials are comprised of a non-plastic base (e.g., concrete or wood) with a thin polymer-based coating. This coating has the potential to determine the observed reflectance of a given pixel, since photons generally only interact with the surficial micron (or less) of such opaque materials. It is critical for end-users to understand this nuance that our map includes non-plastic items coated in plastic.

Another important limitation of the approach is the bias of high MF scores towards pixels with large instances of relatively compositionally homogenous materials. This is also observed in the frequent detection of roofing materials in our study area. While this is useful for users interested in roofing materials and identification/monitoring of very large plastic and/or polymer-coated hotspots, it is suboptimal for users interested in change of fractional cover of waste plastics in sparse mixtures. It is possible that a spectral mixing-based approach might alleviate this issue and is recommended for exploration in future work. Multitemporal imaging is also likely to be useful in this regard, since roofing materials are less likely to change appreciably on short time scales, while dumping of waste is a much more dynamic process.

The accuracy assessment used in this analysis had important limitations. Primary among these limitations was the lag between image collection (2020) and field observations (2021), which was partly due to uncertainty in the timing of WV3 acquisitions, which are not known in advance. Lack of access, time, and human resources while in the field all introduced important constraints on our ability to collect comprehensive and thorough field validation. Further, visual interpretation of the VNIR imagery used for the validation introduced subjectivity and human error. While an unfortunate consequence of data access limitations in a funding-constrained environment, such constraints are likely also faced by many end-users.

#### Future work

Watershed-scale maps of plastics and polymer-coated debris could be useful for waste managers and hydrologists identifying and prioritizing areas with need for intervention. In Los Laureles Canyon Watershed in particular, these results could be improved by pairing the matched filter results with machine learning techniques to optimize the mapping of impacted areas like clandestine dumpsites or other emerging macroplastic aggregations. Providing specific parameters, such as the typical shape and size of plastic waste patches, the mean reflectance spectra of waste patches in WV3 imagery, laboratory convolved measurements for different polymer types, and clues of where waste patches and informal landfills are situated, a machine learning model could be trained to distinguish sparse plastic waste from other materials in complex backgrounds and differentiate these areas from plastic-bearing and/or polymer-coated urban infrastructure.

Other important avenues for future work include detailed examination of the generality of the polymer endmember found in this environment to other similar areas, in-depth comparisons of different unmixing methods (linear spectral unmixing vs. MF), or how characteristic differences in urban watersheds (e.g., prevailing weather conditions, topography, economic development) influence the applicability of the research.

Using these approaches to understand landscape dynamics, including sources and spatiotemporal patterns of waste aggregations, and ultimately estimates of marine debris loading from storm events, is currently limited by WV3 data coverage but expected to grow as more data from WV3 and other sensors become available.

## Conclusions

We identified spectroscopic and multispectral properties of plastics from waste discarded in a complex urban watershed. WV3 imagery successfully mapped areas with high fractional cover of suspected plastic-bearing and polymer-coated materials, such as roofs, courts, and unmanaged waste aggregations. An inferred polymer image endmember correlated closely with laboratory spectra of four distinct types of plastic, and a mean spectrum created from averaging laboratory reflectance measurements of 18 plastics with polymer types: high-density polyethylene (HDPE), low-density polyethylene (LDPE), polyethylene terephthalate (PET), polystyrene (PS), and polyvinyl chloride (PVC) (*r* = 0.95). High separability was observed in the WV3 spectral feature space between the polymer endmember and non-polymer substrates, vegetation, and dark endmembers. Our study suggests that WV3 SWIR imagery of complex urban watersheds may have a characteristic polymer endmember in the spectral feature space and that common plastic polymers like HDPE, LDPE, PVC, and PET may be distinguishable in such study areas using this generalized image endmember. While this model performed well in our study area, additional work is needed to understand generalizability to higher resolution airborne imagery, or in other urban environments which may contain a greater material diversity.

## Electronic supplementary material

Below is the link to the electronic supplementary material.

Supplementary file1 (XLSX 13 KB)

Supplementary file2 (XLSX 105 KB)

Supplementary file3 (DOCX 677 KB)

## Data Availability

Data is provided within the submission as supplementary material and includes laboratory reflectance spectra for 17 field-collected samples and the trash surveys performed in Los Laureles Canyon Watershed to characterize solid waste accumulations and hotspots.

## References

[CR1] Aguilar, E. (2023). Mapping plastic in an urban watershed using laboratory reflectance spectroscopy and WorldView-3 multispectral SWIR imagery. (Master's thesis) San Diego State University San Diego, CA. Retrieved January 2024 from https://digitalcollections.sdsu.edu/do/a9d31086-1a64-4216-b8e5-0d3936aa35f2

[CR2] Asadzadeh, S., & de Souza Filho, C. R. (2016). Investigating the capability of WorldView-3 superspectral data for direct hydrocarbon detection. *Remote Sens Environ,**173*, 162–173. 10.1016/j.rse.2015.11.030

[CR3] Aslam, S., Tzoraki, O., & Krasakopoulou, E. (2022). Anthropogenic litter in freshwater bodies and their estuaries: An empirical analysis in Lesvos, Greece. *Environ Sci Pollut Res,**29*, 16563–16575. 10.1007/s11356-021-16793-z 10.1007/s11356-021-16793-z 34648163

[CR4] Biermann, L., Clewley, D., Martinez-Vicente, V., & Topouzelis, K. (2020). Finding plastic patches in coastal waters using optical satellite data. *Sci Rep,**10*, 5364. 10.1038/s41598-020-62298-z 32327674 10.1038/s41598-020-62298-z PMC7181820

[CR5] Biggs, T. W., Anderson, W. G., & Pombo, O. A. (2014). Concrete and poverty, vegetation and wealth? A counterexample from remote sensing of socioeconomic indicators on the U.S.–Mexico Border. *Prof Geogr,**67*, 166–179. 10.1080/00330124.2014.905161

[CR6] Blettler, M. C., Abrial, E., Khan, F. R., Sivri, N., & Espinola, L. A. (2018). Freshwater plastic pollution: Recognizing research biases and identifying knowledge gaps. *Water Res,**143*, 416–424. 10.1016/j.watres.2018.06.015 29986250 10.1016/j.watres.2018.06.015

[CR7] Blume, S., Franke, J., Garaba, S., Giang, P., Mathis, J., Ortwig, N., & Ziegler, S. (2023). Advances in remote sensing of plastic waste. Deutsche Gesellschaft für Internationale Zusammenarbeit (GIZ) GmbH. Retrieved June 2022 from https://www.giz.de/en/downloads/giz-2023-en-advances-in-remote-sensing-of-plastic-waste.pdf

[CR8] Boardman, J. W. (1998). Leveraging the high dimensionality of AVIRIS data for improved sub-pixel target unmixing and rejection of false positives: mixture tuned matched filtering. *NASA Jet Propulsion Laboratory*, *97*, 55–56. Retrieved June 2022 from: https://www.semanticscholar.org/paper/Leveraging-the-High-Dimensionality-of-AVIRIS-Data-i-Boardman/56b74f1da3746aa51733bf50aec811ad8ca67e07

[CR9] Boardman, J. W., & Kruse, F. A. (2011). Analysis of imaging spectrometer data using $ n $-dimensional geometry and a mixture-tuned matched filtering approach. *IEEE Trans Geosci Remote Sens,**49*, 4138–4152. 10.1109/TGRS.2011.2161585

[CR10] Carvalho Júnior, O. A., Guimarães, R. F., Gillespie, A. R., Silva, N. C., & Gomes, R. A. (2011). A new approach to change vector analysis using distance and similarity measures. *Remote Sens,**3*, 2473–2493. 10.3390/rs3112473

[CR11] Chandrasekhar, S. (1960). *Radiative Transfer*. Dover Publications, Inc.

[CR12] Cordova, M. R., Bernier, N., Yogaswara, D., Subandi, R., Wibowo, S. P. A., Kaisupy, M. T., & Haulussy, J. (2023). Land-derived litter load to the Indian Ocean: A case study in the Cimandiri River, southern West Java, Indonesia. *Environ Monit Assess,**195*, 1251. 10.1007/s10661-023-11831-4 37768383 10.1007/s10661-023-11831-4

[CR13] Elvidge, C. D. (1990). Visible and near infrared reflectance characteristics of dry plant materials. *Int J Remote Sens,**11*, 1775–1795. 10.1080/01431169008955129

[CR14] Garaba, S. P., Aitken, J., Slat, B., Dierssen, H. M., Lebreton, L., Zielinski, O., & Reisser, J. (2018). Sensing ocean plastics with an airborne hyperspectral shortwave infrared imager. *Environ Sci Technol,**52*, 11699–11707. 10.1021/acs.est.8b02855 30249095 10.1021/acs.est.8b02855

[CR15] Garaba, S. P., & Dierssen, H. M. (2018). An airborne remote sensing case study of synthetic hydrocarbon detection using short wave infrared absorption features identified from marine-harvested macro-and microplastics. *Remote Sens Environ,**205*, 224–235. 10.1016/j.rse.2017.11.023

[CR16] Garaba, S. P., & Dierssen, H. M. (2020). Hyperspectral ultraviolet to shortwave infrared characteristics of marine-harvested, washed-ashore and virgin plastics. *Earth Syst Sci Data,**12*, 77–86. 10.5194/essd-12-77-2020

[CR17] Garaba, S. P., Arias, M., Corradi, P., Harmel, T., de Vries, R., & Lebreton, L. (2021). Concentration, anisotropic and apparent colour effects on optical reflectance properties of virgin and ocean-harvested plastics. *J Hazard Mater,**406*, 124290. 10.1016/j.jhazmat.2020.124290 33390286 10.1016/j.jhazmat.2020.124290

[CR18] Geyer, R., Jambeck, J. R., & Law, K. L. (2017). Production, use, and fate of all plastics ever made. *Sci Adv,**3*, e1700782. 10.1126/sciadv.1700782 28776036 10.1126/sciadv.1700782 PMC5517107

[CR19] Gnann, N., Baschek, B., & Ternes, T. A. (2022). Close-range remote sensing-based detection and identification of macroplastics on water assisted by artificial intelligence: A review. *Water Res,**222*, 118902. 10.1016/j.watres.2022.118902 35944407 10.1016/j.watres.2022.118902

[CR20] Goodrich, K. A., Basolo, V., Feldman, D. L., Matthew, R. A., Schubert, J. E., Luke, A., Eguiarte, A., Boudreau, D., Serrano, K., & Reyes, A. S. (2020). Addressing pluvial flash flooding through community-based collaborative research in Tijuana, Mexico. *Water,**12*, 1257. 10.3390/w12051257

[CR21] Goushki, M. N., Shiri, M. A., & Nozari, M. (2023). Estimation of gas emissions using the LandGEM model from the landfill of Baft County, Kerman, Iran. *Environ Monit Assess,**195*, 1444. 10.1007/s10661-023-11943-x 37946053 10.1007/s10661-023-11943-x

[CR22] Grover, R. (2011). *Local perceptions on environmental degradation and community infrastructure in Los Laureles Canyon, Tijuana, Mexico *(Master’s Thesis). San Diego State University, San Diego, CA. Retrieved June 2022 from: https://digitalcollections.sdsu.edu/do/c1a1dc8d-1591-4b42-a7e2-aee37c49b72e/file/d4d998d9-80d1-415f-b937-c6477d508046/download/sdsu:4021%20OBJ%20Datastream.pdf

[CR23] Guha, B., Momtaz, Z., Kafy, A. A., & Rahaman, Z. A. (2023). Estimating solid waste generation and suitability analysis of landfill sites using regression, geospatial, and remote sensing techniques in Rangpur, Bangladesh. *Environ Monit Assess,**195*, 54. 10.1007/s10661-022-10695-4 10.1007/s10661-022-10695-4 36323908

[CR24] Guo, X., & Li, P. (2020). Mapping plastic materials in an urban area: Development of the normalized difference plastic index using WorldView-3 superspectral data. *ISPRS J Photogramm Remote Sens,**169*, 214–226. 10.1016/j.isprsjprs.2020.09.009

[CR25] Gudino-Elizondo, N., Biggs, T. W., Bingner, R. L., Langendoen, E. J., Kretzschmar, T., Taguas, E. V., Taniguchi-Quan, K. T., Liden, D., & Yuan, Y. (2019). Modelling runoff and sediment loads in a developing coastal watershed of the US-Mexico border. *Water,**11*, 1024. 10.3390/w11051024 10.3390/w11051024 PMC6775644 31583124

[CR26] Gudino-Elizondo, N., Brand, M. W., Biggs, T. W., Hinojosa-Corona, A., Gómez-Gutiérrez, Á., Langendoen, E., Bingner, R., Yuan, Y., & Sanders, B. F. (2022). Rapid assessment of abrupt urban mega-gully and landslide events with structure-from-motion photogrammetric techniques validates link to water resources infrastructure failures in an urban periphery. *Nat Hazards Earth Syst Sci,**22*(2), 523–38. 10.5194/nhess-22-523-2022

[CR27] Harsanyi, J. C., & Chang, C.-I. (1994). Hyperspectral image classification and dimensionality reduction: An orthogonal subspace projection approach. *IEEE Trans Geosci Remote Sens,**32*, 779–785. 10.1109/36.298007

[CR28] Heiden, U., Segl, K., Roessner, S., & Kaufmann, H. (2007). Determination of robust spectral features for identification of urban surface materials in hyperspectral remote sensing data. *Remote Sens Environ,**111*, 537–552. 10.1016/j.rse.2007.04.008

[CR29] Hueni, A., & Bertschi, S. (2020). Detection of sub-pixel plastic abundance on water surfaces using airborne imaging spectroscopy. *IEEE*, 6325–6328. 10.1109/IGARSS39084.2020.9323556.

[CR30] Jambeck, J. R., Geyer, R., Wilcox, C., Siegler, T. R., Perryman, M., Andrady, A., ... & Law, K. L. (2015). Plastic waste inputs from land into the ocean. *Science*, *347*(6223), 768–771. 10.1126/science.1260352. 10.1126/science.1260352 25678662

[CR31] Jeffreys, H. (1997). An invariant form for the prior probability in estimation problems. proceedings of the Royal Society of London. Series A. *Math Phys Sci,**186*, 453–461. 10.1098/rspa.1946.0056 10.1098/rspa.1946.0056 20998741

[CR32] Knaeps, E., Sterckx, S., Strackx, G., Mijnendonckx, J., Moshtaghi, M., Garaba, S. P., & Meire, D. (2021). Hyperspectral-reflectance dataset of dry, wet and submerged marine litter. *Earth Syst Sci Data,**13*, 713–730. 10.5194/essd-13-713-2021

[CR33] Kokaly, R., Clark, R., Swayze, G., Livo, K., Hoefen, T., Pearson, N., Wise, R., Benzel, W., Lowers, H., & Driscoll, R. (2017). USGS spectral library version 7 data: US geological survey data release. United States Geological Survey (USGS). 10.3133/ds1035.

[CR34] Kruse, C., Boyda, E., Chen, S., Karra, K., Bou-Nahra, T., Hammer, D., et al. (2023). Satellite monitoring of terrestrial plastic waste. *PLoS ONE,**18*(1), e0278997. 10.1371/journal.pone.0278997 36652417 10.1371/journal.pone.0278997 PMC9847976

[CR35] Kuester, M. (2016). Radiometric Use of WorldView-3 Imagery (Technical Note). *Digital Globe*. Retrieved from: https://dgv4-cms-827production.s3.amazonaws.com/uploads/document/file/142/Radiometric_Use_of_WorldView-3_v2.pdf

[CR36] Lebreton, L. C., Van Der Zwet, J., Damsteeg, J. W., Slat, B., Andrady, A., & Reisser, J. (2017). River plastic emissions to the world’s oceans. *Nat Commun,**8*(1), 15611. 10.1038/ncomms15611 28589961 10.1038/ncomms15611 PMC5467230

[CR37] Livens, S., Knaeps, E., Benhadj, I., Bomans, B., & Dries, J. (2022). Feasibility assessment of a dedicated satellite mission for monitoring marine macroplastics. 4S symposium ResearchGate.

[CR38] Lopez-Galvez, N. I. L. (2014) Soil analysis of organic and inorganic contaminants in Goat Canyon (Cañon De Los Laureles), at the US-Mexico border. (Master's thesis) San Diego State University San Diego, CA. Retrieved June 2022 from https://digitalcollections.sdsu.edu/do/ae33af65-7448-4302-a214-01b79aab1a51

[CR39] Mac Arthur, A., MacLellan, C. J., & Malthus, T. (2012). The fields of view and directional response functions of two field spectroradiometers. *IEEE Trans Geosci Remote Sens,**50*, 3892–3907. 10.1109/TGRS.2012.2185055

[CR40] Masoumi, H., Safavi, S. M., & Khani, Z. (2012). Identification and classification of plastic resins using near infrared reflectance. *International Journal of Mechanical Engineering*, *6, *213–220.

[CR41] Martínez-Vicente, V., Clark, J. R., Corradi, P., Aliani, S., Arias, M., Bochow, M., Bonnery, G., Cole, M., Cózar, A., & Donnelly, R. (2019). Measuring marine plastic debris from space: Initial assessment of observation requirements. *Remote Sens,**11*, 2443. 10.3390/rs11202443

[CR42] Maximenko, N., Corradi, P., Law, K. L., Van Sebille, E., Garaba, S. P., Lampitt, R. S., Galgani, F., Martinez-Vicente, V., Goddijn-Murphy, L., & Veiga, J. M. (2019). Toward the integrated marine debris observing system. *Front Mar Sci,**6*, 447. 10.3389/fmars.2019.00447

[CR43] Meijer, L. J., Van Emmerik, T., Van Der Ent, R., Schmidt, C., & Lebreton, L. (2021). More than 1000 rivers account for 80% of global riverine plastic emissions into the ocean. *Sci Adv,**7*, e5803. 10.1126/sciadv.aaz5803 10.1126/sciadv.aaz5803 PMC8087412 33931460

[CR44] Moore, S., Hale, T., Weisberg, S., Flores, L., & Kauhanen, P. (2020). California trash monitoring methods and assessments playbook. SFEI Contribution No. 1025. Richmond, CA: San Fransicso Estuary Institute.

[CR45] Moshtaghi, M., Knaeps, E., Sterckx, S., Garaba, S., & Meire, D. (2021). Spectral reflectance of marine macroplastics in the VNIR and SWIR measured in a controlled environment. *Sci Rep,**11*, 5436. 10.1038/s41598-021-84867-6 33686150 10.1038/s41598-021-84867-6 PMC7940656

[CR46] Neigh, C. S., Masek, J. G., & Nickeson, J. E. (2013). High-resolution satellite data open for government research. *Eos Trans Am Geophys Union,**94*, 121–123. 10.1002/2013EO130002

[CR47] Pandit, M. K., & Kateja, A. (2023). Hydrochemistry and groundwater quality assessment around solid waste landfill sites in peri-urban Jaipur, NW India. *Environ Monit Assess,**195*, 557. 10.1007/s10661-023-11128-6 37043144 10.1007/s10661-023-11128-6

[CR48] Park, Y.-J., Garaba, S. P., & Sainte-Rose, B. (2021). Detecting the great pacific garbage patch floating plastic litter using WorldView-3 satellite imagery. *Opt Exp,**29*, 35288–35298. 10.1364/OE.440380 10.1364/OE.440380 34808966

[CR49] PlasticsEurope. (2022). Plastics– the Facts 2022. *PlasticsEurope*, *1*, 1-82. Retrieved from: https://plasticseurope.org/knowledge-hub/plastics-the-facts-2022/

[CR50] Richards, J. A. & Jia, X. (1999). *Remote Sensing Digital Image Analysis*. Springer Berlin, Heidelberg. 10.1007/978-3-662-03978-6

[CR51] Scheffler, D., Hollstein, A., Diedrich, H., Segl, K., & Hostert, P. (2017). AROSICS: An automated and robust open-source image co-registration software for multi-sensor satellite data. *Remote Sens,**9*(7), 676. 10.3390/rs9070676

[CR52] Schmidt, C., Krauth, T., & Wagner, S. (2017). Export of plastic debris by rivers into the sea. *Environ Sci Technol,**51*(21), 12246–12253. 10.1021/acs.est.7b02368 29019247 10.1021/acs.est.7b02368

[CR53] Schmidt, T., Kuester, T., Smith, T., & Bochow, M. (2023). Potential of optical spaceborne sensors for the differentiation of plastics in the environment. *Remote Sens,**15*(8), 2020. 10.3390/rs15082020

[CR54] Small, C., & Sousa, D. (2022). The Sentinel 2 MSI spectral mixing space. *Remote Sens*. 10.3390/rs14225748

[CR55] Sousa, D., & Small, C. (2017). Global cross-calibration of landsat spectral mixture models. *Remote Sens Environ,**192*, 139–149. 10.1016/j.rse.2017.01.033

[CR56] Sousa, D., & Small, C. (2022). Joint characterization of Sentinel-2 reflectance: Insights from manifold learning. *Remote Sens,**14*(22), 5688. 10.3390/rs14225688

[CR57] Tasseron, P., Van Emmerik, T., Peller, J., Schreyers, L., & Biermann, L. (2021). Advancing floating macroplastic detection from space using experimental hyperspectral imagery. *Remote Sens,**13*, 2335. 10.3390/rs13122335

[CR58] Tasseron, P. F., Schreyers, L., Peller, J., Biermann, L., & van Emmerik, T. (2022). Toward robust river plastic detection: Combining lab and field-based hyperspectral imagery. *Earth Space Sci,**9*, e2022EA002518. 10.1029/2022EA002518

[CR59] Thenkabail, P., GangadharaRao, P., Biggs, T., Krishna, M., & Turral, H. (2007). Spectral matching techniques to determine historical land-use/land-cover (LULC) and irrigated areas using time-series 0.1-degree AVHRR Pathfinder Datasets. *Photogrammetric Engineering & Remote Sensing, 73*, 1029–1040.

[CR60] Thuillier, G., Herse, M., Labs, D., Foujols, T., Peetermans, W., Gillotay, D., Simon, P. C., & Mandel, H. (2003). The solar spectral irradiance from 200 to 2400 nm as measure by the SOLSPEC spectrometer from The atlas and Eureca Missions. *Solar Physics,* *214*, 1–22.

[CR61] Turin, G. (1960). An introduction to matched Filters. *IRE Trans Inf Theory,**6*, 311–329. 10.1109/TIT.1960.1057571

[CR62] van Emmerik, T., & Schwarz, A. (2020). Plastic debris in rivers. Wiley interdisciplinary reviews. *Water,**7*, e1398. 10.1002/wat2.1398

[CR63] Wagner, M., Monclús, L., Arp, H. P. H., Groh, K. J., Løseth, M. E., Muncke, J., Wang, Z., Wolf, R., & Zimmermann, L. (2024). State of the science on plastic chemicals - Identifying and addressing chemicals and polymers of concern. PlastChem. 10.5281/zenodo.10701706.

[CR64] Woodward, P. M. (1951). Information theory and the design of radar receivers. *Proc IRE,**39*, 1521–1524. 10.1109/JRPROC.1951.273638

[CR65] Zhou, S., Kuester, T., Bochow, M., Bohn, N., Brell, M., & Kaufmann, H. A. (2021). Knowledge-based, validated classifier for the identification of aliphatic and aromatic plastics by WorldView-3 satellite data. *Remote Sens Environ,**264*, 112598. 10.1016/j.rse.2021.112598

