# Supplementary material for: Mapping terrestrial macroplastics and polymer-coated materials in an urban watershed using WorldView-3 and laboratory reflectance spectroscopy
Source: Environ Monit Assess. 2025 Jun 25;197(7):802. doi: 10.1007/s10661-025-14125-z (PMC12198079; doi:10.1007/s10661-025-14125-z)
Supplement: Supplementary file 3 — Supplementary file3 (DOCX 677 KB) [file 10661_2025_14125_MOESM3_ESM.docx]

Mapping terrestrial macroplastics and polymer-coated materials in an urban watershed using WorldView-3 and laboratory reflectance spectroscopy

Elena Aguilar ^1^, Daniel Sousa ^1^, Amy V. Uhrin ^2^, Napoleon Gudino-Elizondo ^3^, and Trent Biggs ^1,^ *

^1^ Department of Geography, San Diego State University, San Diego, California 92182, United States; [eaguilar0070@sdsu.edu](mailto:eaguilar0070@sdsu.edu); [dan.sousa@sdsu.edu](mailto:dan.sousa@sdsu.edu); [tbiggs@sdsu.edu](mailto:tbiggs@sdsu.edu)

^2^ National Oceanic and Atmospheric Administration, National Ocean Service, Office of Response and Restoration, Marine Debris Division, Silver Spring, Maryland, 20910, United States; [amy.uhrin@noaa.gov](mailto:amy.uhrin@noaa.gov)

^3^ Instituto de Investigaciones Oceanológicas, Universidad Autónoma de Baja California, Ensenada, Baja California 22760, México; [ngudino@uabc.edu.mx](mailto:ngudino@uabc.edu.mx)

***** Correspondence: Prof. Trent Biggs, E-mail: [tbiggs@sdsu.edu](mailto:tbiggs@sdsu.edu), Prof. Dan Sousa, E-mail: [dan.sousa@sdsu.edu](mailto:dan.sousa@sdsu.edu);

**Supplementary Material**

**Table S1.** WorldView-3 SWIR center wavelengths (Kuester, 2016).

| **Spectral Band** | **Center Wavelength** | **Effective Bandwidth Δλ (nm)** |
| --- | --- | --- |
| SWIR 1 | 1209.1 | 33.0 |
| SWIR 2 | 1571.6 | 39.7 |
| SWIR 3 | 1661.1 | 37.3 |
| SWIR 4 | 1729.5 | 41.6 |
| SWIR 5 | 2163.7 | 38.9 |
| SWIR 6 | 2202.2 | 40.9 |
| SWIR 7 | 2259.3 | 47.6 |
| SWIR 8 | 2329.2 | 67.9 |


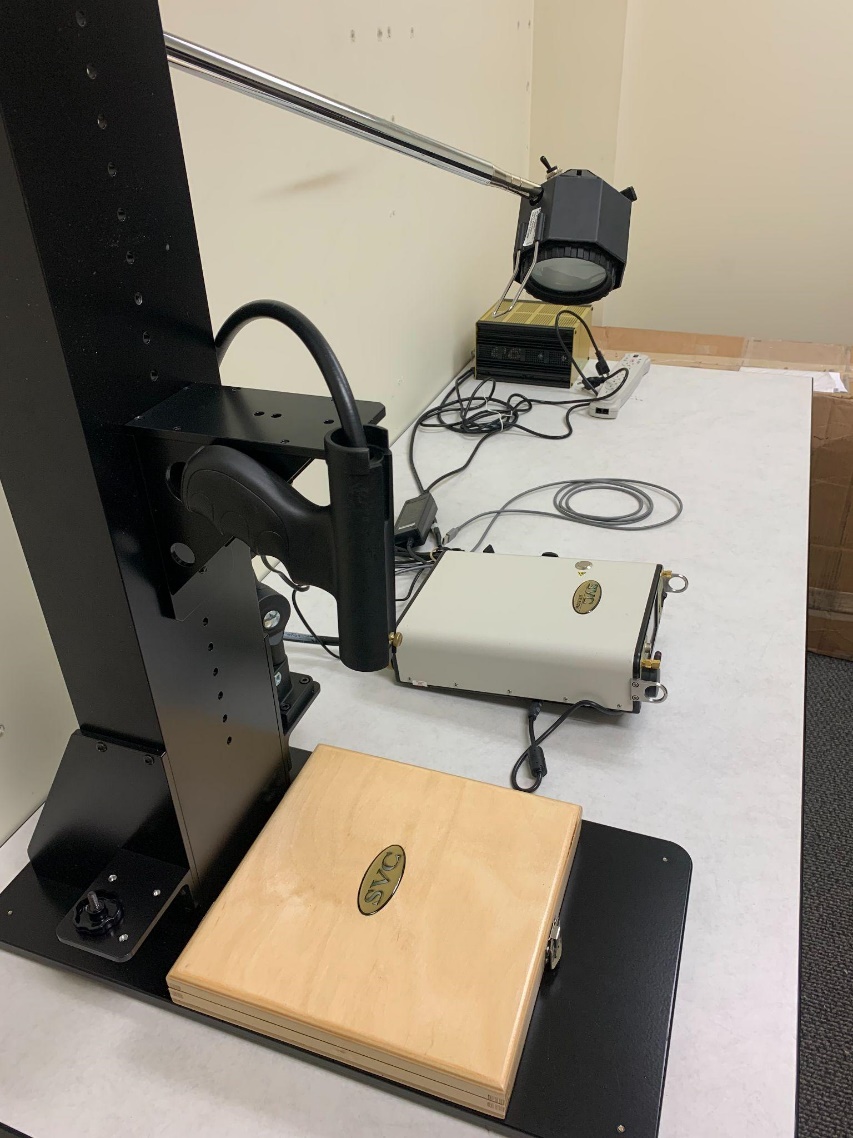


**Fig. S1** Laboratory set up. SVC spectroradiometer (white box) is connected to a measurement pistol that is mounted 16 inches above the Spectralon®, located in the foreground. The tungsten-halogen Sunnex lamp is placed about two feet away and at a 60◦ angle and from the waste items being measured.


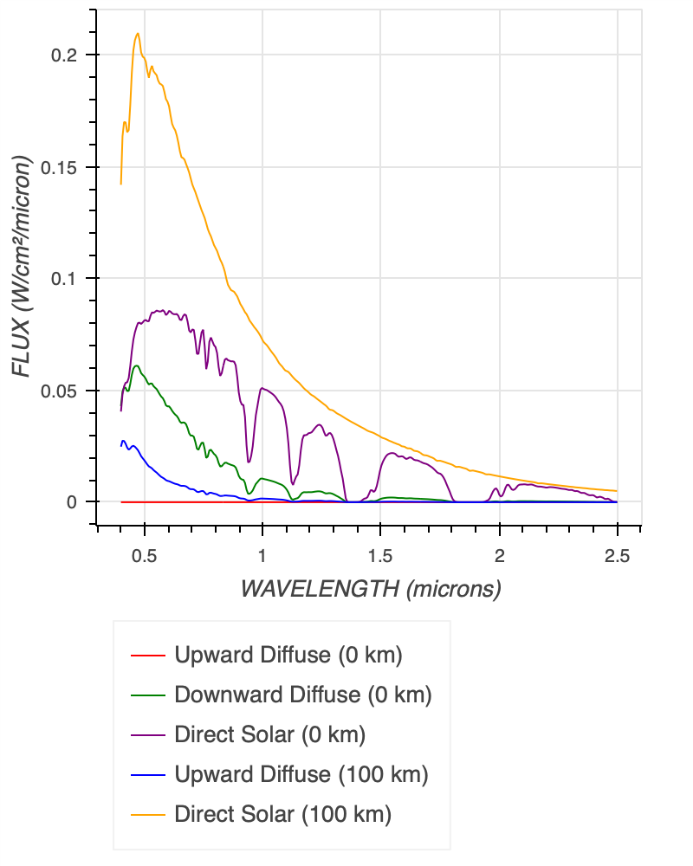

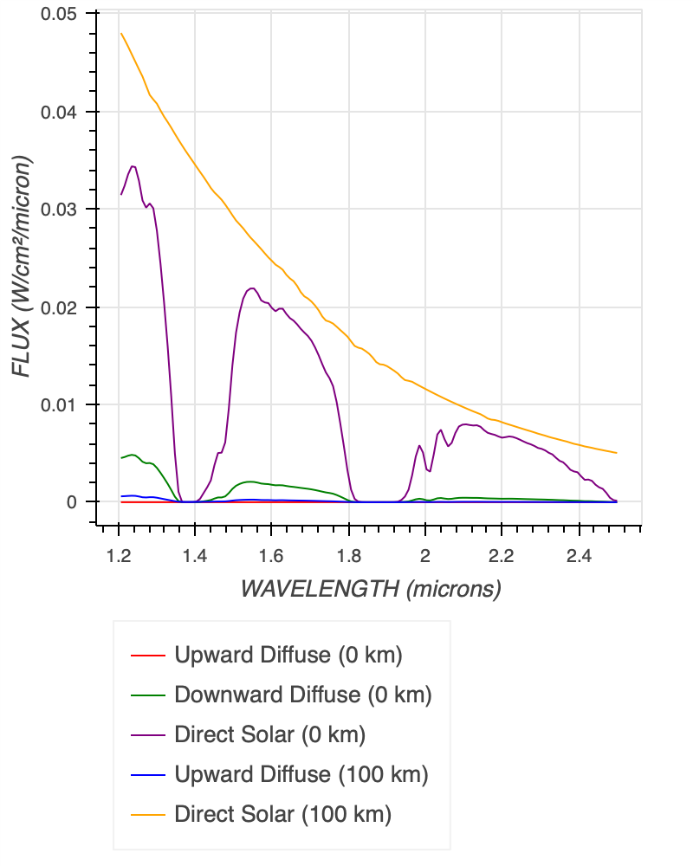


**Fig. S2** MODTRAN-based forward model of top-of-atmosphere direct solar radiant flux (goldenrod) relative to the top-of-atmosphere upward diffuse radiant flux (blue). Diffuse radiant flux includes Rayleigh scattering, as well as other potential scattering modes. Important parameters used for this run include a mid-latitude summer atmosphere, urban aerosol model, visibility of 10 km (in accord with Tijuana Airport data record). Both the full VSWIR spectral range (left) and the range of the WorldView-3 SWIR bands (right) are shown. As expected from first principles, the diffuse scattering profile is dominated by Rayleigh scattering, which is strongly inversely wavelength-dependent. The strongest impact is modeled in the UV and visible blue, and weakest impact in the SWIR bands which are the focus of this study. Absolute values of diffuse radiant flux are 4.93 x 10^-4^ and 1.77 x 10^-5^ at 1200 and 2400 nm, respectively. Absolute values of direct solar radiant flux are 4.76 x 10^-2^ and 6.02 x 10^-2^ at 1200 and 2400 nm. The impact of this effect is thus expected to be somewhere between 1.04% and 0.30% of the overall signal. Interested readers are directed to <http://modtran.spectral.com/modtran_home> for further exploration


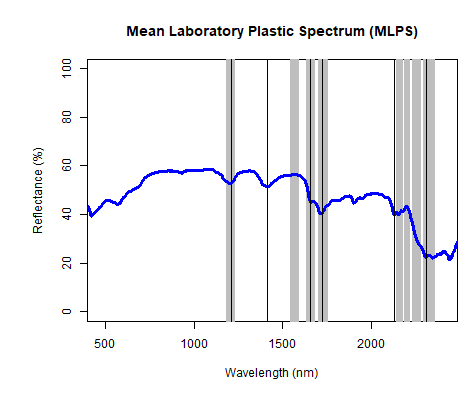


**Fig. S3** Mean laboratory plastic spectrum. Gray vertical lines indicate the position of SWIR bands 1-8. Black thin vertical lines indicate the position at which common absorptions occur across the 5 polymers (the last two black lines represent a larger range from 2132-2313).


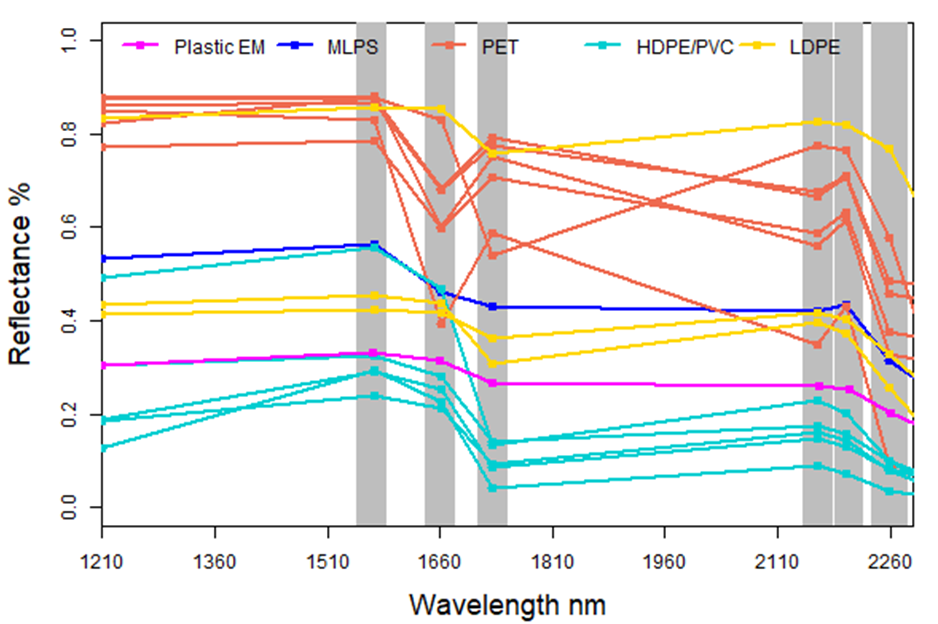


**Fig. S4** The image polymer endmember (EM; magenta) looks most similar to PET (orange), HDPE/PVC (cyan), LDPE (yellow), and the mean laboratory plastic spectrum (MLPS) (blue).
